# A hypomorphic model of CPS1 deficiency for investigating the effects of hyperammonemia on the developing nervous system

**DOI:** 10.1242/dmm.052303

**Published:** 2025-06-20

**Authors:** Stuti Bakshi, Taryn Diep, Brandon J. Willis, Rachel Reyes, Grace F. Wu, Georgios Makris, Martin Poms, Isabel Day, Qin Sun, Irina Zhuravka, Lindsay Lueptow, Michelle Tang, Gareth A. Cromie, Aimée M. Dudley, Johannes Häberle, Gerald S. Lipshutz

**Affiliations:** ^1^ Departments of Surgery, David Geffen School of Medicine at UCLA, Los Angeles, CA 90095, USA; ^2^ Mouse Biology Program, University of California, Davis, Davis, CA 95618, USA; ^3^ Division of Metabolism and Children's Research Center, University Children's Hospital Zurich, Zurich 8008, Switzerland; ^4^ Clinical Chemistry and Biochemistry, University Children's Hospital Zurich, Zurich 8008, Switzerland; ^5^ Molecular and Medical Pharmacology, David Geffen School of Medicine at UCLA, Los Angeles, CA 90095, USA; ^6^ Department of Molecular and Human Genetics, Baylor College of Medicine, Houston, TX 77030, USA; ^7^ Department of Psychology, UCLA, Los Angeles, CA 90095, USA; ^8^ Pacific Northwest Research Institute, Seattle, WA 98122, USA; ^9^ Molecular Biology Institute, David Geffen School of Medicine at UCLA, Los Angeles, CA 90095, USA; ^10^ Psychiatry, David Geffen School of Medicine at UCLA, Los Angeles, CA 90095, USA; ^11^ Intellectual and Developmental Disabilities Research Center at UCLA, David Geffen School of Medicine at UCLA, Los Angeles, CA 90095, USA; ^12^ Semel Institute for Neuroscience, David Geffen School of Medicine at UCLA, Los Angeles, CA 90095, USA

**Keywords:** Carbamoyl phosphate synthetase 1 deficiency, Human mutation, Enzyme activity, Hyperammonemia, Glutamine

## Abstract

Carbamoyl phosphate synthetase 1 (CPS1) deficiency is a rare metabolic disorder that, in neonatal onset, is typically characterized by severe life-threatening and neurologically injuring hyperammonemic episodes with high unmet patient need. Patients that retain limited enzyme activity may present later in life with less severe hyperammonemia. CPS1 drives the first step in the urea cycle, the pathway terrestrial mammals utilize to metabolize nitrogen. In order to probe the effect of hyperammonemia on the developing nervous system and explore new therapies, a murine *Cps1* exon 3-4 mutant was previously generated. However, these mice die within 24 h of birth, limiting study capabilities. Herein, we developed a novel *Cps1* hypomorphic murine model with residual enzyme activity that maintains survival, but with dysfunction of Cps1 that could be detected biochemically. Characterization, based on the orthologous human variant Asn674Ile, revealed that the variant is reproducible, 100% penetrant and biochemically phenocopies the human disorder. The hypomorph presents with elevated ammonia and glutamate, and reduced citrulline, and with an impaired rate of ureagenesis, providing a novel platform to study and develop therapies for CPS1 deficiency.

## Teaser

**Summary:** A hypomorphic mouse model of carbamoyl phosphate synthetase 1 deficiency was developed, allowing assessment of the effects of mild hyperammonemia on development, with potential use in new therapeutic advancements.

## INTRODUCTION

Carbamoyl phosphate synthetase 1 (CPS1) deficiency is a monogenic autosomal disorder caused by variants in CPS1, a proximal urea cycle enzyme. It is one of the least common urea cycle disorders, with an estimated prevalence of 1 in 300,000-1,000,000 live births, although likely an underestimation (Diez-Fernandez and Haberle, 2017; Nagata et al., 1991; Summar et al., 2013). CPS1 catalyzes the first step in the cycle, metabolizing nitrogen through converting ammonia into non-toxic urea (Summar, 1998). The trademark manifestation of the neonatal-onset form of the disorder is rapidly progressive metabolic encephalopathy presenting in the first few days after birth and subsequent severe life-threatening/neurologically injuring recurrent episodes of hyperammonemia (Diez-Fernandez and Haberle, 2017; Walker, 2014). Patients that retain some enzyme activity may present later (Ishikawa et al., 2022) with less severe hyperammonemia. However, recurrences of metabolic decompensation and, in general, chronically but mildly elevated ammonia, is associated with diminished mental ability (Waisbren et al., 2019). Although the unmet need is high, and traditional treatment options for preventing hyperammonemia are limited, preclinical results for *CPS1* gene therapy suggest some promise (Nitzahn et al., 2020; Diep et al., 2024).

In order to improve understanding of the disorder and advance gene-based therapies, mouse models have been developed. The first (Schofield et al., 1999), as a biallelic mutant, reproduced the neonatal-onset form with hyperammonemia shortly after birth and death within 36 h; this model was not submitted to a repository and was lost. Our group more recently produced a neonatal-onset murine model with excision of floxed exons 3-4 (Khoja et al., 2019), reproducing the phenotype: marked hyperammonemia/hyperglutaminemia and death with 24 h. Both models have limited use (i.e. early/rapid increase in ammonia, small size, early death) in understanding the effect of hyperammonemia on the development of the early nervous system and in advancing new therapeutic approaches.

To develop a larger model of the disorder, our group advanced the use of Cre-lox technology, developing a murine model in which exons 3 and 4 were floxed. Over the course of 3 weeks after initiating *Cps1* genomic recombination, hyperammonemia and hyperglutaminemia ensue, with weight loss, sarcopenia, derangement of amino acids and, ultimately, death (Khoja et al., 2018). Although such mice can be rescued with an adenoviral vector (Khoja et al., 2018) or dual-vector adeno-associated virus approach (Nitzahn et al., 2020), the method to achieve this model is artificial and not truly representative of the human clinical condition, whether neonatal onset or as a later presentation.

There is a need for a mouse model that phenocopies CPS1 deficiency without the technical and biological hurdles associated with the severe neonatal-onset or delayed-onset Cre recombination. To advance new therapeutic approaches and understand the effects of chronic hyperammonemia on the developing nervous system, we sought to develop a *Cps1* hypomorph. This model would be capable of overcoming the deficiencies of those previously developed while phenocopying mild to moderate deficiency. The requirements were to generate a knock-in (KI) murine model in which adult size could be achieved, some residual endogenous Cps1 activity was present, and it simultaneously had at least mild hyperammonemia and amino acid derangements. We describe herein the successful development and characterization of such a murine model based on the human Asn674Ile variant, reproducing the biochemical hallmarks of the disorder.

## RESULTS

### The Asn674Ile variant is predicted to alter CPS1 function

CPS1 Asn674Ile was predicted to be deleterious by four variant effect predictors (Table S1). We examined the distribution of amino acid variations across organisms at that position and found that Ile is completely absent from the multiple sequence alignment of 50,951 CPS1 orthologs from EVE [Protein Data Bank (PDB) structural identifier 2eve] (Table S2). Moreover, other amino acids with hydrophobic side chains were either absent or under-represented in the EVE multiple sequence alignment of CPS1 orthologs, suggesting that variants with hydrophobic side chains are under purifying selection.

**Fig. 1.**
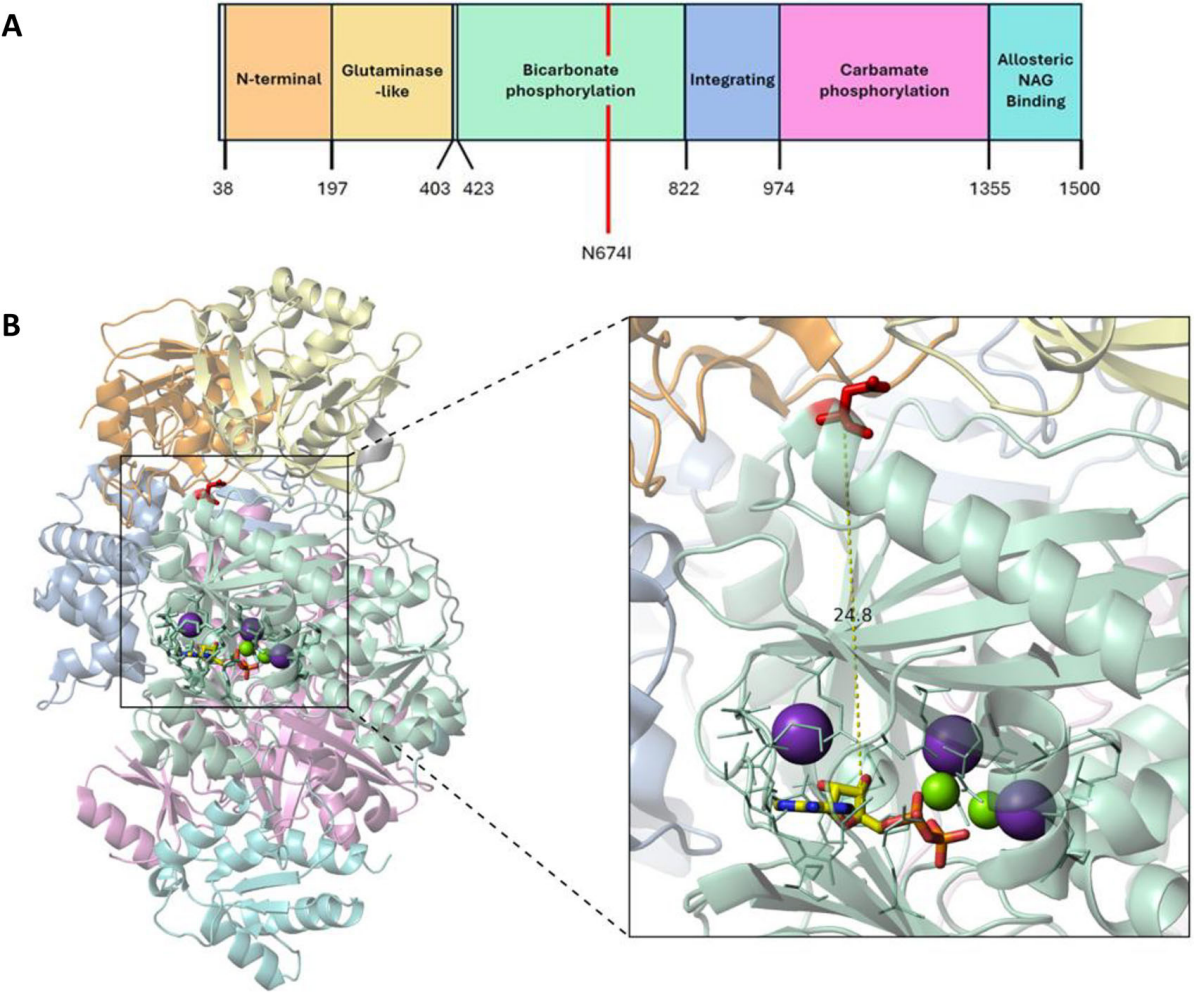
**The Asn674Ile CPS1 variant is in the bicarbonate phosphorylation region and converts a polar uncharged amino acid to one that is nonpolar and hydrophobic.** (A) The domain composition is noted and the amino acid location is identified by the numerals. The mutation is in the C-terminal moiety with bicarbonate phosphorylation, the first two steps in the production of carbamoyl phosphate by phosphorylation of bicarbonate by ATP to produce carboxyphosphate followed by attack by NH_3_ to yield carbamate [adapted from de Cima et al. (2015) under the terms of the CC-BY 4.0 license]. (B) Cartoon representation and enlargement (inset) of the ligand-bound human CPS1 protein (PDB ID, 5DOU; de Cima et al., 2015) transparency set to 50%. (Note that colors of the domain correspond with those used in A.) Asn674 is highlighted in red sticks. ADP in the bicarbonate phosphorylation domain is shown in red, orange, yellow and blue sticks. K^+^ and Mg^2+^ ions are shown in purple and green spheres, respectively. Catalytic residues around ADP are shown in light green lines. The yellow dashed line indicates the distance between the center of the Asn674 residue and the center of ADP in angstroms.

We also used AlphaFold3 to evaluate the functional effect of Asn674Ile on the predicted CPS1 protein structures. Although Asn674 is found in the bicarbonate phosphorylation domain of CPS1, it is situated outside the active center in which the first phosphorylation occurs and carbamate is formed (de Cima et al., 2015) (Fig. 1A,B). Although structural predictions are limited in their ability to capture protein dynamics, structural alignments between wild type (WT) and Asn674Ile variant models for both human and murine CPS1 revealed very little change in overall protein fold and arrangement [template modeling score>0.99 for superposition of WT CPS1 and Asn674Ile variant for both human and murine; root mean square deviation (RMSD)=0.42 for superposition of 1323 alpha-carbons in human WT CPS1 and human CPS1 Asn674Ile; RMSD=0.191 for superposition of 1203 alpha-carbons in murine WT Cps1 and murine Asn674Ile Cps1]. Superposition of the bicarbonate phosphorylation domain of the human Asn674Ile model to the corresponding domain in the solved ligand-bound human CPS1 structure also revealed little change in architecture and orientation (RMSD=0.325 of superposition of 369 alpha-carbons in human CPS1 Asn674Ile and human CPS1 WT; PDB ID, 5DOU). Variant analysis in SpliceAI revealed scores of 0 and 0.1, predicting that there is a very low probability that the variation will result in acceptor gain/loss and donor gain/loss (i.e. scores<0.2). Thus, the variant is very unlikely to affect splicing and lead to a truncated protein. In summary, the variant effect and structural predictions of CPS1 Asn674Ile variant, together with the evidence from the case report of the mild CPS1 deficiency seen in a patient with this variant (Kurokawa et al., 2007), suggested that CPS1 Asn674Ile would be a mild pathogenic variant.

### Asn674Ile Cps1 variant results in postnatal survival without evidence of liver pathology

Successful pregnancies from brother/sister inbreeding of heterozygous *Cps1*^+/N674I^ mice to produce homozygosity were performed. Both male and female homozygotes were viable and fertile and maintained as an inbred homozygous strain (*Cps1*^N674I/N674I^) on the C57BL/6 background. Mice were bred to heterozygous *Cps1* null (exon 3-4 mutant) mice (Khoja et al., 2019) to produce compound heterozygotes (*Cps1*^0/N674I^). These were also viable and fertile. Litters did not demonstrate a preference for one sex, and there was no evidence of intrauterine or early postnatal death in either the homozygote or compound heterozygote, with no obvious difference in size or in cage activity.

**Fig. 2.**
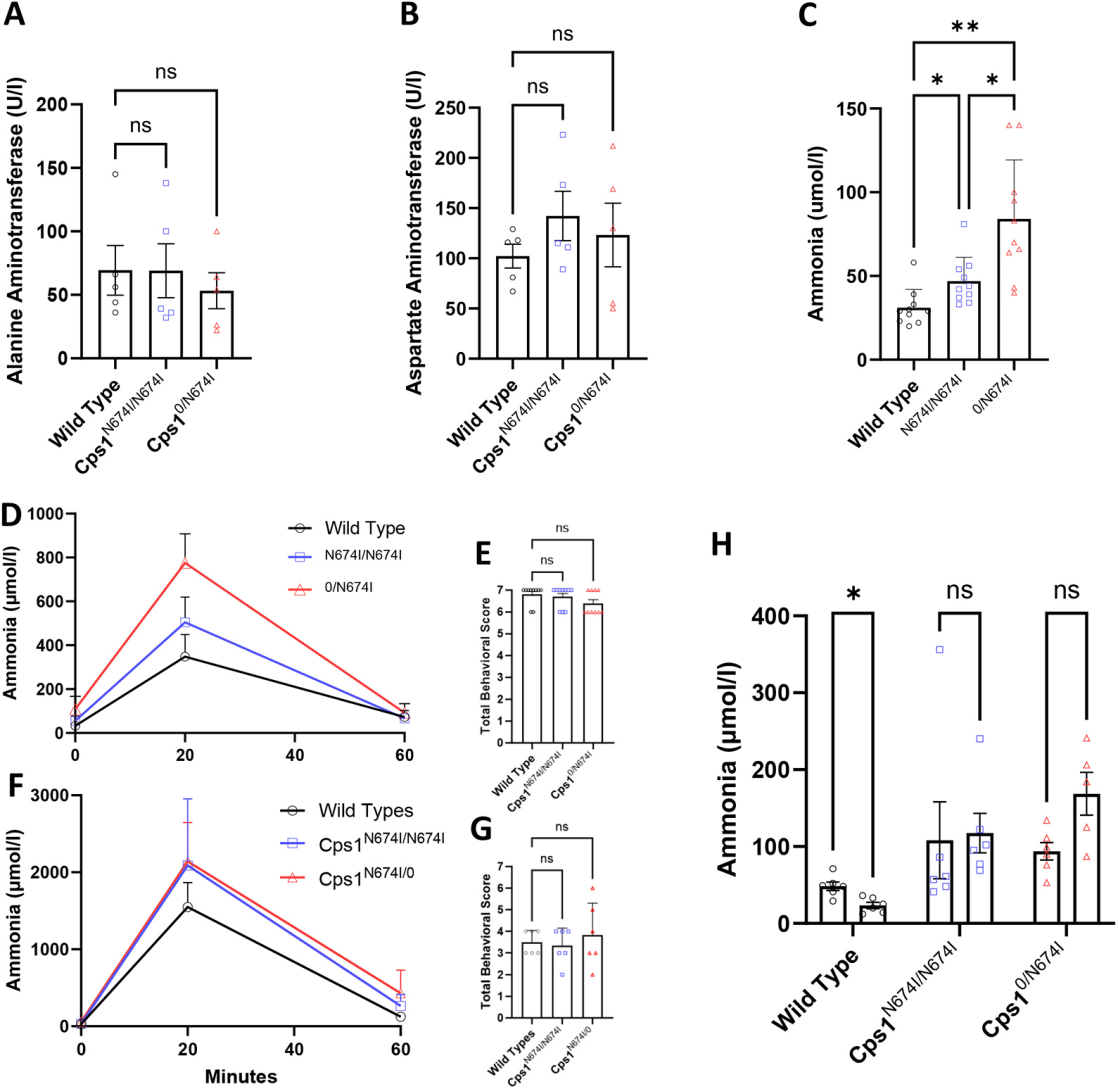
**Cps1 hypomorphic mouse models demonstrate normal liver enzymes while having elevated ammonia and altered response to ammonia loading and carglumic acid administration.** (A,B) Values for the liver transaminases alanine aminotransferase and aspartate aminotransferase from plasma of both *Cps1*^N674I/N674I^ and *Cps1*^0/N674I^ hypomorphic mouse models were similar to wild-type values. (C) Plasma ammonia has a step-wise increase based on the Cps1 mutation, with wild type<*Cps1*^N674I/N674I^<*Cps1*^0/N674I^: 31.00±10.99 µm/l, 46.90±14.35 µm/l and 84.10±35.26 µm/l, respectively. (D) Ammonium challenging with 5 mmol/kg demonstrates a step-wise increase in plasma ammonia based on genotype at 20 min after administration and declines close to baseline at 60 min (*n*=10 per group). (E) Behavioral testing was performed 15 min after administration. (F) When a higher dose of ammonium is administered (7.5 mmol/kg), greater differences are detected between the hypomorphic and wild-type mice. (G) Behavioral scores were similar between groups reflective of the effect of high blood ammonia levels on the central nervous system. (H) Carglumic acid in drinking water led to a decline in plasma ammonia in wild-type mice but did not in the hypomorphic mice. *n*=4-5 per group for A,B, *n*=10 per group for D-F, *n*=6 per group for G,H, and *n*=5-6 per group for I. Data presented as mean±s.d. ns, not significant; *P<0.05, **P<0.01 (A-G, one-way ANOVA with Dunnett's T3 multiple comparison test; H, paired two-tailed *t*-test).

Transaminase levels were determined in age-matched mice of both sexes examining for biochemical evidence of liver pathology. Alanine aminotransferase (ALT) (Fig. 2A) from plasma was similar between WT, Asn671Ile homozygotes (*Cps1*^N674I/N674I^) and null/Asn674Ile mice (*Cps1*^0/N674I^) (*n*=5 per group) (*P*=0.99, WT vs homozygote; *P*=0.76, WT vs compound heterozygote). Similarly, there was no statistically significant difference in aspartate aminotransferase (AST) (Fig. 2B) levels (*n*=5 per group) (*P*=0.33, WT vs homozygote; *P*=0.79, WT vs compound heterozygote).

### Asn674Ile mutation in mice results in mild hyperammonemia, and the compound heterozygote results in further increase in blood ammonia

Ammonia was measured from whole blood of WT, *Cps1*^N674I/N674I^ and *Cps1*^0/N674I^ mice (*n*=10 per group; five males, five females) (Fig. 2C) on standard mouse chow (20% protein). Levels were found to have a step-wise increase based on the mutation, with WT<*Cps1*^N674I/N674I^<*Cps1*^0/N674I^: 31.00±10.99 µmol/l, 46.90±14.35 µmol/l and 84.10±35.26 µmol/l, respectively. Differences in ammonia levels were statistically significant (*P*=0.037, WT vs *Cps1*^N674I/N674I^; *P*=0.0024, WT vs *Cps1*^0/N674I^; *P*=0.027, *Cps1*^N674I/N674I^ vs *Cps1*^N674I/0^).

Mice were challenged with ammonium chloride to assess response and urea cycle function (Fig. 2D), administering 5 mmol/kg by intraperitoneal route (*n*=6 per group; three males, three females) and determining blood ammonia immediately before administration and 20 and 60 min thereafter. There were differences in starting (i.e. baseline) ammonia levels: WT, 32.90±14.85 µmol/l; *Cps1*^N674I/N674I^, 55.10±22.31 µmol/l; *Cps1*^0/N674I^, 108.90±58.35 µmol/l (WT vs *Cps1*^N674I/N674I^, *P*=0.036; WT vs *Cps1*^0/N674I^, *P*=0.005). At 20 min, peak measured ammonia levels were determined: WT, 348.00±100.45 µmol/l; *Cps1*^N674I/N674I^, 504.80±114.65 µmol/l; *Cps1*^0/N674I^, 775.60±132.35 µmol/l (WT vs *Cps1*^N674I/N674I^, *P*=0.009; WT vs *Cps1*^0/N674I^, *P*<0.0001). At 60 min, ammonia was at or near pre-injection levels: WT, 73.10±28.80 µmol/l; *Cps1*^N674I/N674I^, 66.00±12.11 µmol/l; *Cps1*^0/N674I^, 92.40±41.30 µmol/l (WT vs *Cps1*^N674I/N674I^, *P*=0.728; WT vs *Cps1*^0/N674I^, *P*=0.420). A three-measure behavioral test was performed 15 min after ammonium chloride administration. Behavioral testing (Fig. 2E) did not demonstrate differences between groups [scored entries: ataxia (0-2), seizure activity (0-2) and response to sound (0-3) (Ye et al., 1997)].

We performed ammonium challenging again with 7.5 mmol/kg of ammonium chloride, finding that the response was greater in all groups (*n*=6 per group; three males, three females) (Fig. 2F). Baseline blood ammonia levels were as follows: WT, 28.33±9.33 µmol/l; *Cps1*^N674I/N674I^, 33.17±13.62 µmol/l; *Cps1*^0/N674I^, 59.67±23.13 µmol/l (WT vs *Cps1*^N674I/N674I^, *P*=0.7308; WT vs *Cps1*^0/N674I^, *P*=0.034). At 20 min, peak ammonia levels were measured: WT, 1548.33±318.71 µmol/l; *Cps1*^N674I/N674I^, 2088.33±866.10 µmol/l; *Cps1*^0/N674I^, 2140.00±507.15 µmol/l (WT vs *Cps1*^N674I/N674I^, *P*=0.346; WT vs *Cps1*^0/N674I^, *P*=0.079). At 60 min, blood ammonia returned to near pre-injection levels in WT but remained elevated in the hypomorphs; however, these differences were trending to but not statistically significant: WT, 123.33±40.33µmol/L; *Cps1*^N674I/N674I^, 264.83±149.64 µmol/l; *Cps1*^0/N674I^, 428.67±300.51 µmol/l (WT vs *Cps1*^N674I/N674I^, *P*=0.122; WT vs *Cps1*^0/N674I^, *P*=0.103). Behavioral testing (Fig. 2G) again did not demonstrate significant differences between the groups, but mice were more greatly affected than with lower-dose ammonium.

Response to carglumic acid was examined (Fig. 2H). Mice (generally *n*=6 per group; three males, three females) had reverse osmosis water replaced with water containing 5% dextrose/0.1% carglumic acid for 1 week prior to testing. WT demonstrated a decline in ammonia from 43.88±13.86 µmol/l to 23.33±9.83 µmol/l (*P*=0.039). Cps1 mutant mice demonstrated no statistically significant response.

### *Cps1* RNA expression is increased, and protein analyses demonstrate a reduction in protein and enzymatic activity

**Fig. 3.**
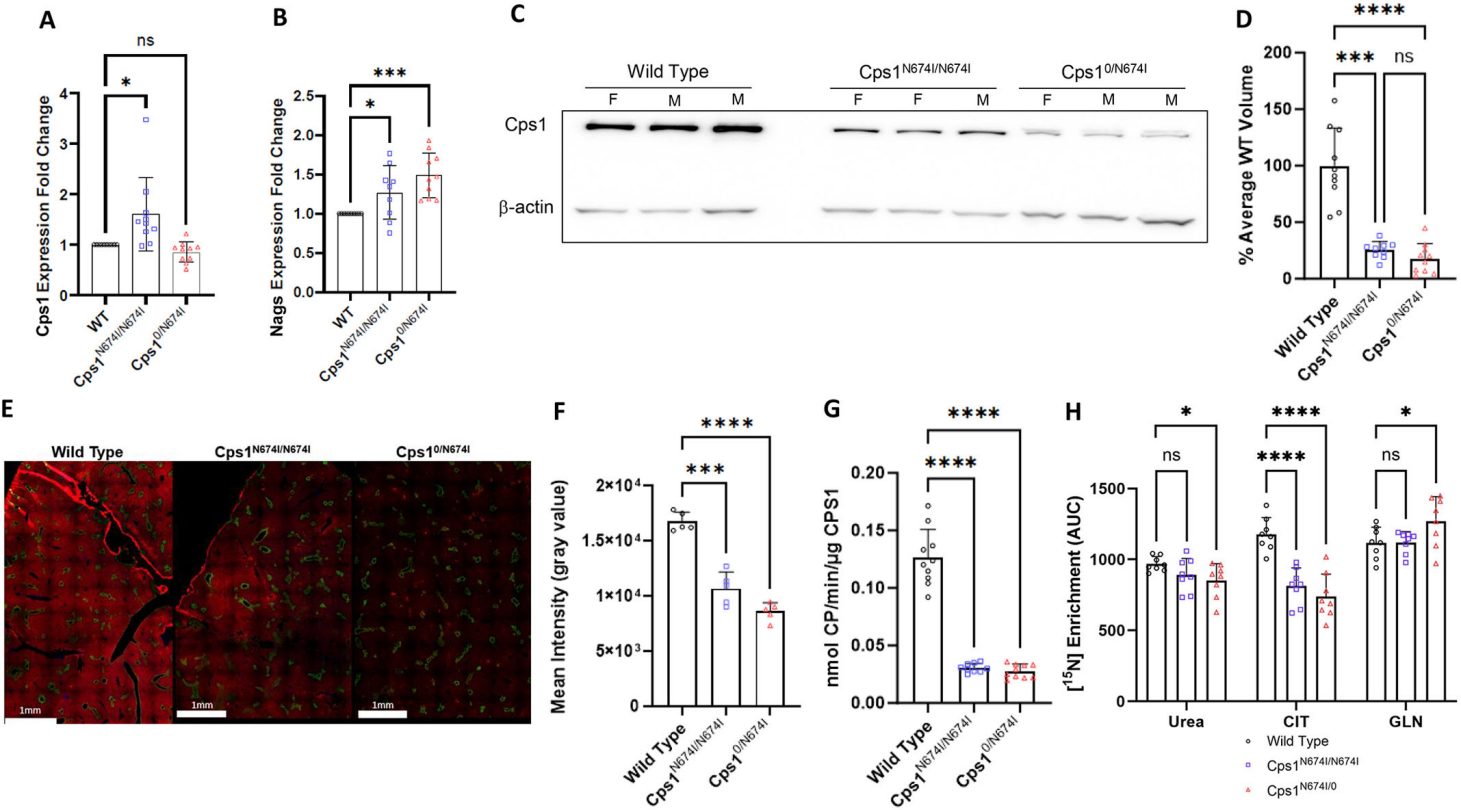
**Cps1 hypomorphic models demonstrate increased *Nags* expression and reduced Cps1 protein and enzyme activity, while ureagenesis is maintained.** (A) Fold change in hepatic *Cps1* RNA expression comparing genotypes was performed normalized to *Cps1* wild-type mice (at 1). (B) Fold change in hepatic *Nags* RNA expression was determined comparing genotypes normalized to *Nags* wild-type mice (at 1). (C) Representative western blot images from different mouse genotypes examining hepatic Cps1 expression with β-actin loading control (each lane represents a different mouse). (D) Quantitation of western blot of Cps1 protein levels between genotypes demonstrating markedly reduced protein in the hypomorphic mice. (E,F) Representative Cps1 liver immunostaining of the different mouse genotypes (Cps1 in red, glutamine synthetase in green) (E), with average fluorescence intensity per group represented (F). (G) Cps1 enzyme activity was determined from each genotype, showing marked reduction in the hypomorphic mouse livers. (H) [^15^N]urea enrichment is decreased in the compound heterozygote, while [^15^N]citrulline enrichment is decreased in both the homozygous mutant and the compound heterozygote. [^15^N]glutamine is increased in the compound heterozygote. *n*=10 per group for A,B,D,G; *n*=8 per group for H; *n*=3 mice per group for E,F with ROI as *n*=5 (see Fig. S2). ns, not significant; *P<0.05, ***P<0.001, ****P<0.0001 (A,B,F,G,H, one-way ANOVA with Dunnett's T3 multiple comparison test; D, ordinary one-way ANOVA Tukey's multiple comparison test). AUC, area under the curve; CIT, citrulline; F, female; GLN, glutamine; M, male; WT, wild type. Scale bars: 1 mm.

Quantitative real-time PCR was performed to determine whether *Cps1* expression was increased in hepatocytes of hypomorphic mice (Fig. 3A). With WT *Cps1* mice (*n*=10) standardized at 1.0 *Cps1* RNA expression, *Cps1*^N674I/N674I^ (*n*=9) demonstrated increased *Cps1* RNA expression (1.614±0.725-fold upregulation), whereas *Cps1*^0/N674I^ (*n*=10) demonstrated reduced *Cps1* RNA expression (0.861±0.201-fold downregulation). N-acetylglutamate synthase (*Nags*) was upregulated in both groups: 1.269±0.340-fold increase in *Cps1*^N674I/N674I^ (*n*=9), and 1.489±0.283-fold increase in *Cps1*^0/N674I^, compared to that in WT mice (Fig. 3B). Cps1 protein was reduced in both the homozygous and compound heterozygote compared to that in WT mice (Fig. 3C,D). Quantitatively, Cps1 band density by western blotting was 25.4% of that of WT for *Cps1*^N674I/N674I^ and 17.9% of that of WT for *Cps1*^0/N674I^. Cps1 immunostaining (Fig. 3E) revealed differences in visualized intensity of Cps1 protein between WT, *Cps1*^N674I/N674I^ and *Cps1*^0/N674I^. Quantitation of absolute fluorescence intensity [*n*=3 per group; five regions of interest (ROIs) of liver per mouse] demonstrated greatest mean intensity in the WT (16,754.0±850.1), followed by *Cps1*^N674I/N674I^ (10,693.0±1492.0) and *Cps1*^0/N674I^ (8605.0±811.0) (Fig. 3F). Cps1 liver enzymatic activity (*n*=9-10 livers per group) (Fig. 3G) revealed WT mice at 0.1263±0.0244 nmol carbamoyl phosphate (CP)/min/µg Cps1, while enzymatic activity in *Cps1*^N674I/N674I^ was 0.03054±0.00383 nmol CP/min/µg Cps1 (24.18% of WT) (*P*<0.0001) and further reduced in *Cps1*^0/N674I^ mice at 0.02775±0.00188 nmol CP/min/µg Cps1 (21.97%) (*P*<0.0001). Ureagenesis with 20% protein mouse chow was decreased in both *Cps1*^N674I/N674I^ and *Cps1*^0/N674I^ mice (Fig. 3H). Compared to that in WT mice, [^15^N] incorporation was slightly reduced in *Cps1*^N674I/N674I^ (8% reduction; *P*=0.1403), with a significant further reduction in *Cps1*^0/N674I^ (total 12.0% reduction; *P*=0.0304). Both *Cps1*^N674I/N674I^ and *Cps1*^0/N674I^ mice showed significantly decreased [^15^N] incorporation in citrulline (31% reduction, *P*<0.00001 and 37.4% reduction, *P*<0.00001, respectively), while *Cps1*^0/N674I^ mice additionally exhibited increased [^15^N] incorporation into glutamine (13% increase, *P*=0.033).

### Plasma amino acid analysis demonstrate elevated glutamate and other amino acid alterations

**Fig. 4.**
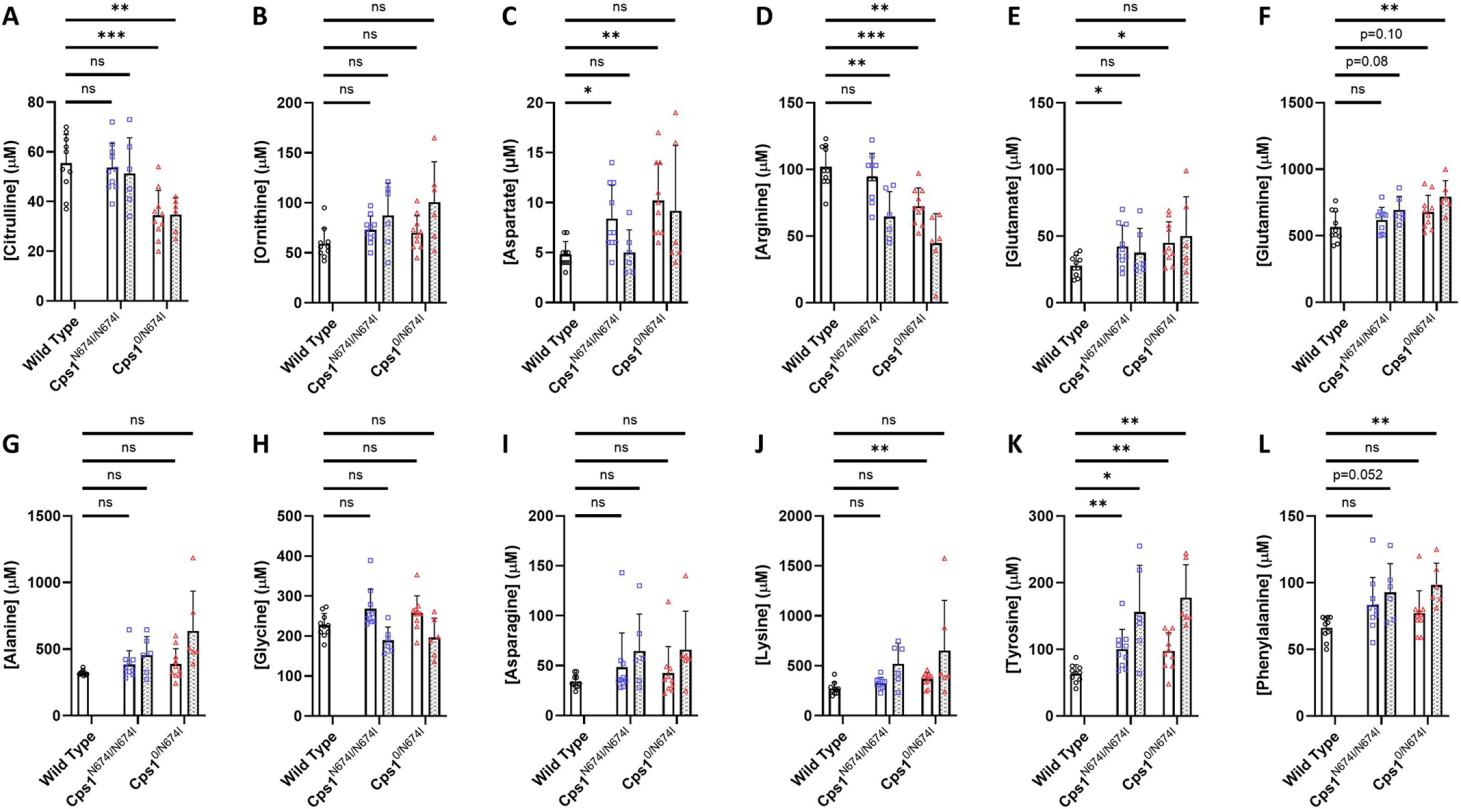
**Plasma amino acids show derangements in urea cycle-related amino acids.** (A-L) Plasma from adult mice of each genotype was examined for concentration of urea cycle-related amino acid analyte when receiving standard mouse chow with 20% protein (white bars) and when receiving mouse chow with 50% protein (spiculated bars): citrulline (A), ornithine (B), aspartate (C), arginine (D), glutamate (E), glutamine (F), alanine (G), glycine (H), asparagine (I), lysine (J), tyrosine (K) and phenylalanine (L). *n*=10 per group for standard protein chow; *n*=6 per group for high-protein chow. Data are presented in µM and as mean±s.d. ns, not significant; *P<0.05, **P<0.01, ***P<0.001 (A-L, one-way ANOVA with Dunnett's T3 multiple comparison test).

Plasma was collected for amino acid analysis, with mice receiving standard mouse chow (20% protein) (Fig. 4, white bars). This was followed by Cps1 hypomorphic mice receiving high-protein chow (52% protein) for 2 weeks and plasma collection (Fig. 4, spiculated bars). A number of primary derangements were found that have likely secondary or downstream effects on intermediates. In general (but with a few exceptions), amino acid profile changes were more pronounced in the compound heterozygous compared to the homozygous mutant and were also generally more pronounced when mice received the high-protein chow.

Citrulline (Fig. 4A) was reduced in the compound heterozygote on both regular chow (*P*=0.0008 vs WT) (WT, 55.40±11.59; *Cps1*^N674I/N674I^, 53.70±9.72; *Cps1*^0/N674I^, 34.50±9.98) and high protein (*P*=0.0011 vs WT) (*Cps1*^N674I/N674I^, 51.33±14.38; *Cps1*^0/N674I^, 34.83±6.82), a hallmark finding in CPS1 deficiency as production of carbamoyl phosphate is impaired. This results in less carbamoyl phosphate being available for the next step in the urea cycle, in which it combines with ornithine to form citrulline via ornithine transcarbamoylase (Walker, 2014). Although ornithine (Fig. 4B) was slightly increased, as might be expected, this was not statistically significant with either regular or high-protein chow. Aspartic acid (Fig. 4C) is involved in the next step in the urea cycle by combining with citrulline to form argininosuccinate (Walker, 2014). With less citrulline, argininosuccinate synthesis is limited and aspartate is not efficiently utilized, accumulating in our experimental mice both with regular [WT, 4.80±1.32; *Cps1*^N674I/N674I^, 8.40±3.34 (*P*=0.016 vs WT); *Cps1*^0/N674I^, 10.20±3.74 (*P*=0.002 vs WT)] and high-protein chow, albeit less so. This presumed decrease in synthesis in argininosuccinate would subsequently result in a decrease in arginine (Fig. 4D) synthesis, which was found to be present in these mice when receiving regular chow [WT, 102.10±15.63; *Cps1*^N674I/N674I^, 94.50±17.54 (*P*=0.53 vs WT); *Cps1*^0/N674I^, 72.30±13.37 (*P*=0.0005 vs WT)] and more pronounced with high-protein chow [*Cps1*^N674I/N674I^, 64.67±18.76 (*P*=0.0052 vs WT); *Cps1*^0/N674I^, 44.67±22.12 (*P*=0.0010 vs WT)].

To offset the rise in ammonia, nitrogen is shunted, resulting in specific alterations in certain amino acids that can serve as buffers (Goldsworthy et al., 1968). Glutamate (Fig. 4E) would be expected to increase with elevated ammonia with synthesis from alpha-ketoglutarate through transamination [standard mouse chow: WT, 27.90±7.64; *Cps1*^N674I/N674I^, 42.30±15.84 (*P*=0.044 vs WT); *Cps1*^0/N674I^, 45.10±15.71 (*P*=0.016 vs WT); high protein mouse chow: no statistically significant differences]. If ammonia was markedly elevated, glutamate depletion could occur, with a marked increase in glutamine; with only the mild increase in ammonia with Asn674Ile mutation, only mild, non-statistically significant increases in glutamine (Fig. 4F) in the homozygous mice were detected, with a trend towards significance in the compound heterozygote [WT, 565.90±116.10; *Cps1*^N674I/N674I^, 618.30±94.87 (*P*=0.48 vs WT); *Cps1*^0/N674I^, 678.30±126.40 (*P*=0.10 vs WT)]. This trend increased further and was statistically significant when compound heterozygote mice received high-protein chow [*Cps1*^N674I/N674I^, 693.00±103.80 (*P*=0.0815 vs WT); *Cps1*^0/N674I^, 792.8±121.70 (*P*=0.0084 vs WT)].

CPS1 deficiency may lead to altered alanine and glycine in response to hyperammonemia. Likely owing to limited increases in ammonia in these mouse models, increases in plasma alanine (Fig. 4G) and glycine (Fig. 4H) were also limited. Asparagine (Fig. 4I), the role of which in ammonia detoxification is less direct, can be affected by increases in aspartate and glutamine; however, levels were not statistically different, likely owing to the limited increase in ammonia levels. Further non-statistically significant increases were found with high-protein chow.

Plasma lysine (Fig. 4J) can be increased as a secondary consequence of the disruption of urea cycle functioning (Lukkarinen et al., 2000). Lysine and other amino acids share common transporters and metabolic pathways that may be altered with ammonia accumulation (Mans et al., 1983). Elevated lysine showed a trend toward statistical significance (*P*=0.11) in the homozygous mutant and was statistically significant in the compound heterozygote [WT, 272.80±61.47 vs *Cps1*^0/N674I^, 366.30±67.68 (*P*=0.009 vs WT)]. Further increases in lysine occurred when mice received high-protein chow; however, these were likely not statistically significant owing to the broad standard deviation [*Cps1*^N674I/N674I^, 519.50±206.10 (*P*=0.054 vs WT); *Cps1*^0/N674I^, 651.30±502.60 (*P*=0.221 vs WT)].

Finally, there was alteration in aromatic amino acid metabolism. Tyrosine and phenylalanine can be elevated owing to hyperammonemia-induced catabolism and nitrogen-induced alterations in their metabolism (Yamamoto, 1990). In this mouse model, tyrosine (Fig. 4K) was increased significantly with regular mouse chow [WT, 63.60±13.41; *Cps1*^N674I/N674I^, 100.30±29.72 (*P*=0.007 vs WT); *Cps1*^0/N674I^, 97.70±27.41 (*P*=0.007 vs WT)]; with high-protein chow, these differences increased further [*Cps1*^N674I/N674I^, 156.20±69.93 (*P*=0.044 vs WT); *Cps1*^0/N674I^, 177.80±49.28 (*P*=0.005 vs WT)]. Phenylalanine (Fig. 4L) had a marked trend toward significance in its increase in the homozygous mutant receiving standard chow [WT, 66.30±8.76; *Cps1*^N674I/N674I^, 83.40±20.66 (*P*=0.06 vs WT)] but less so in the compound heterozygote (*P*=0.17 vs WT); however, with high protein mouse chow, the mean plasma phenylalanine values increased in both hypomorphic models [*Cps1*^N674I/N674I^, 92.83±21.53 (*P*=0.052 vs WT); *Cps1*^0/N674I^, 98.33±16.39 (*P*=0.006 vs WT)].

### Hepatic amino acids show more limited derangement

Analysis of intracellular hepatic amino acids showed more limited derangements; little reference values exist, unfortunately, for hepatic amino acid levels in CPS1 deficiency in general, but values have been previously reported for a constitutive Cps1 mutant model (see fig. 4 in Khoja et al., 2019). As with the plasma amino acids, where present, hepatic amino acid abnormalities were (but not exclusively) more pronounced in the compound heterozygote.

**Fig. 5.**
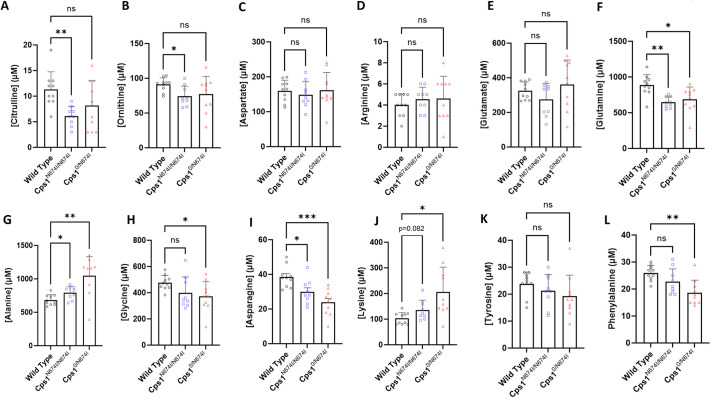
**Hepatic amino acids show some derangements in nitrogen-scavenging amino acids.** (A-L) Hepatic lysates from adult mice of each genotype were examined for concentration of each amino acid analyte: citrulline (A), ornithine (B), aspartate (C), arginine (D), glutamate (E), glutamine (F), alanine (G), glycine (H), asparagine (I), lysine (J), tyrosine (K) and phenylalanine (L). *n*=10 per group). Data are presented in µM and as mean±s.d. ns, not significant; *P<0.05, **P<0.01, ***P<0.001 (A-L, one-way ANOVA with Dunnett's T3 multiple comparison test).

Citrulline (Fig. 5A) was reduced in the homozygous mutant (*P*=0.002 vs WT) (WT, 11.30±3.47; *Cps1*^N674I/N674I^, 6.11±1.90), as was ornithine (Fig. 5B) (*P*=0.02 vs WT) (WT, 91.00±9.74; *Cps1*^N674I/N674I^, 74.33±14.35). Aspartate (Fig. 5C) and arginine (Fig. 5D) were not significantly different between groups.

With ammonia buffering, glutamate was similar between groups (Fig. 5E), whereas glutamine (Fig. 5F) was reduced [WT, 885.00±148.00; *Cps1*^N674I/N674I^, 646.20±77.09 (*P*=0.001 vs WT); *Cps1*^0/N674I^, 686.7±174.30 (*P*=0.026 vs WT)]. As intrahepatic alanine (Fig. 5G) was increased [WT, 684.50±78.12; *Cps1*^N674I/N674I^, 794.00±97.64 (*P*=0.033 vs WT); *Cps1*^0/N674I^, 1047.00±283.30 (*P*=0.006 vs WT)], both glycine (Fig. 5H) [WT, 475.60±55.26; *Cps1*^N674I/N674I^, 398.00±122.2 (*P*=0.198 vs WT); *Cps1*^0/N674I^, 371.20±113.20 (*P*=0.04 vs WT)] and asparagine (Fig. 5I) [WT, 38.50±6.13; *Cps1*^N674I/N674I^, 29.89±6.99 (*P*=0.02 vs WT); *Cps1*^0/N674I^, 23.80±0.39 (*P*=0.0003 vs WT)] were reduced. As with plasma, intrahepatic lysine (Fig. 5J) was increased [WT, 102.80±3.09; *Cps1*^N674I/N674I^, 135.90±38.44 (*P*=0.082 vs WT); *Cps1*^0/N674I^, 206.50±96.80 (*P*=0.016 vs WT)]. Finally, although tyrosine (Fig. 5K) in the *Cps1* mutant mice was not statistically different from that in the WT mice, phenylalanine (Fig. 5L) was reduced in the compound heterozygote [WT, 25.90±2.81; *Cps1*^0/N674I^, 18.60±4.67 (*P*=0.0014 vs WT)].

### *Cps1* hypomorphic mice demonstrate a mild anxiety phenotype

Behavioral phenotyping was performed at 8 months of age (*n*=15, WT; *n*=17, *Cps1*^N674I/N674I^; *n*=12 *Cps1*^0/N674I^). The SHIRPA primary screen examines physical appearance, locomotion and sensory reflex reactions. It was performed to characterize the general phenotype of hypomorphic mice compared to WT. Of the 45 measures, there were only two statistically different findings in the *Cps1*^N674I/N674I^ mice from WT, but six were altered in *Cps1*^0/N674I^ mice (Table 1). As SHIRPA is a screening tool that can miss more subtle changes in cognition or social behavior, more specific testing was performed in other areas to examine for task-specific behavioral changes. These SHIRPA findings did not suggest the presence of global brain dysfunction.

**Table 1.** SHIRPA dominant phenotype mouse screening

	Wild type	*Cps1* ^N674I/N674I^	*P*-value	*Cps1* ^0/N674I^	*P*-value
Viewing jar					
Body position	4.00±0.00	4.00±0.00	>0.99	4.00±0.00	>0.99
Spontaneous activity	1.87±0.74	1.53±0.62	0.32	1.54±0.88	0.51
Respiration rate	2.00±0.00	2.06±0.24	0.44	2.00±0.00	>0.99
Tremor	0.00±0.00	0.12±0.49	0.66	0.20±0.56	0.35
Urination	0.00±0.00	0.00±0.00	>0.99	0.00±0.00	>0.99
Defecation	1.13±1.25	1.71±1.80	0.50	3.23±1.54	<0.01
Arena					
Transfer arousal	4.93±0.26	4.71±0.59	0.29	4.15±1.14	0.06
Locomotor activity	26.27±5.24	26.76±4.35	0.95	21.54±8.19	0.17
Palpebral closure	0.00±0.00	0.00±0.00	>0.99	0.00±0.00	>0.99
Piloerection	0.00±0.00	0.06±0.24	0.65	0.08±0.28	0.53
Gait	0.00±0.00	0.06±0.24	0.65	0.08±0.28	0.53
Pelvic elevation	2.00±0.00	1.94±0.24	0.81	2.08±0.49	0.73
Tail elevation	1.00±0.00	1.12±0.33	0.33	0.92±0.28	0.64
Startle response	1.00±0.00	1.00±0.00	>0.99	0.92±0.28	0.30
Touch escape	2.20±0.56	2.29±0.59	0.87	1.92±0.86	0.55
Held by tail					
Positional passivity	0.00±0.00	0.06±0.24	0.65	0.08±0.28	0.53
Trunk curl	0.93±0.26	0.88±0.33	0.86	0.77±0.44	0.43
Trunk curl on approach	0.07±0.26	0.00±0.00	0.58	0.08±0.28	0.99
Limb grasping	1.00±0.00	1.00±0.00	>0.99	1.00±0.00	>0.99
Abnormal behavior	1.00±0.00	1.00±0.00	>0.99	1.00±0.00	>0.99
Visual placing	2.80±0.56	3.06±0.66	0.28	4.00±0.00	<0.0001
Whisker brush	2.27±0.70	2.06±0.66	0.63	2.54±0.78	0.56
Whisker placement	2.33±0.49	1.94±0.25	0.02	2.54±0.52	0.50
Reflex					
Grip strength	1.67±0.72	1.53±0.80	0.85	1.46±0.66	0.68
Body tone	1.00±0.00	1.06±0.24	0.73	1.15±0.38	0.20
Pinna reflex	1.20±0.41	1.29±0.47	0.80	1.31±0.63	0.84
Corneal reflex	1.00±0.00	1.06±0.24	0.73	1.15±0.38	0.20
Toe pinch	3.20±0.56	2.71±0.92	0.14	2.92±0.95	0.60
Wire maneuver	0.00±0.00	0.56±1.15	0.22	1.39±1.33	<0.01
Supine restraint					
Body length	9.28±0.71	9.47±0.60	0.67	9.62±0.71	0.40
Skin color	1.00±0.00	1.00±0.00	>0.99	0.92±0.28	0.30
Heart rate	1.07±0.26	1.06±0.24	>0.99	0.77±0.44	0.09
Limb tone	1.13±0.35	1.41±0.71	0.30	2.08±0.86	<0.01
Abdominal tone	1.00±0.00	1.00±0.00	>0.99	1.00±0.00	>0.99
Lacrimation	0.33±0.49	0.14±0.44	0.80	0.08±0.28	0.18
Salivation	0.00±0.00	0.00±0.00	>0.99	0.00±0.00	>0.99
Provoked biting	1.00±0.00	1.00±0.00	>0.99	1.00±0.00	>0.99
Penlight vision	1.00±0.00	1.00±0.00	>0.99	1.00±0.00	>0.99
Other					
Righting reflex	0.80±0.41	0.53±0.51	0.21	0.46±0.52	0.14
Contact righting reflex	1.00±0.00	1.00±0.00	>0.99	1.00±0.00	>0.99
Negative geotaxis	1.27±0.96	1.29±0.99	>0.99	0.62±0.96	0.16
Fear	0.13±0.35	0.24±0.44	0.82	0.54±0.52	0.05
Irritability	0.47±0.52	0.35±0.49	0.78	0.69±0.48	0.42
Aggression	0.80±0.41	0.53±0.51	0.21	0.62±0.51	0.51
Vocalization	0.87±0.35	0.47±0.51	0.03	0.38±0.51	0.02

**Fig. 6.**
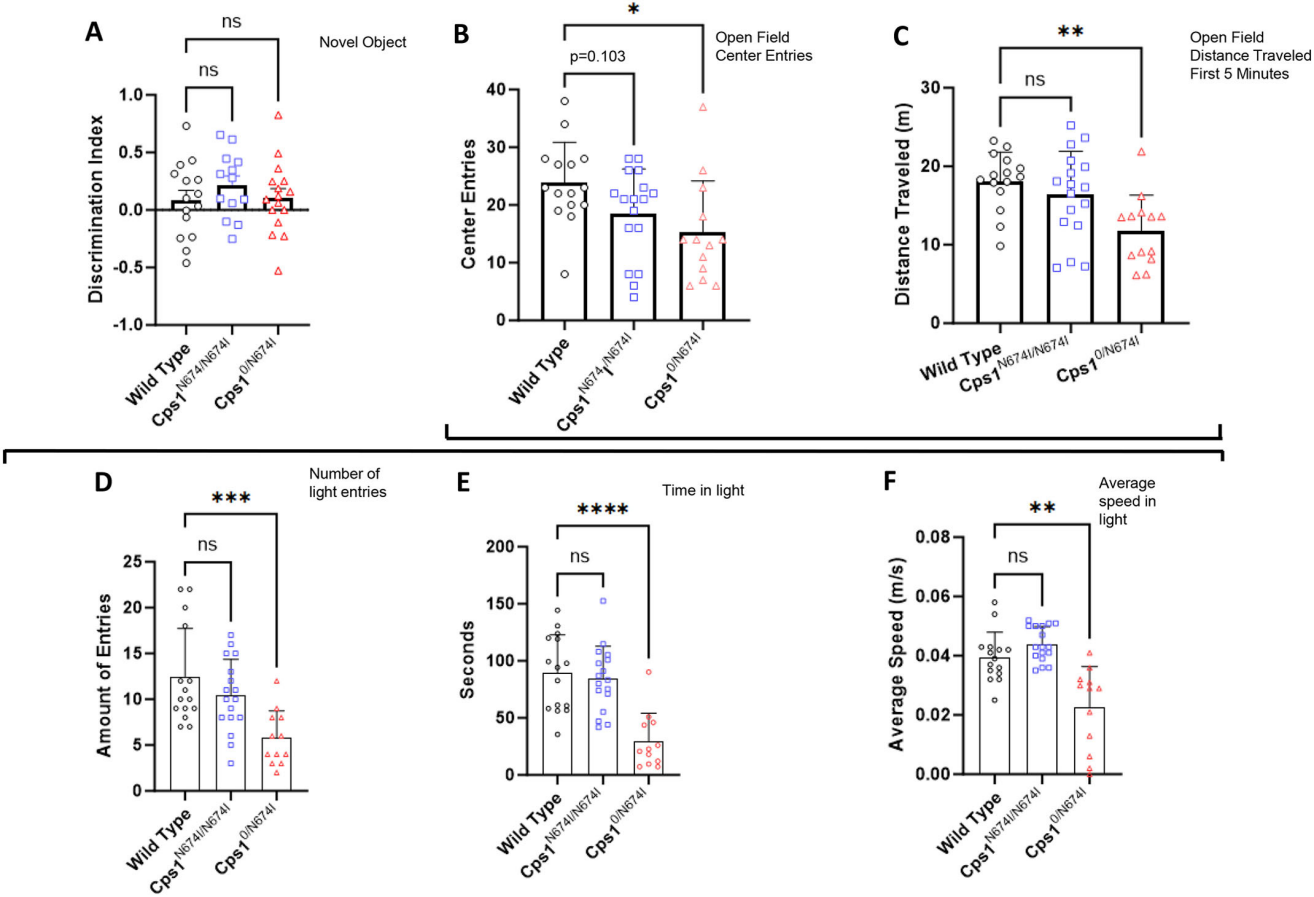
**Behavioral testing of hypomorphic compound heterozygote receiving standard mouse chow reveals an anxiety-like phenotype.** Behavioral phenotype testing was performed on adult mice of each genotype (*n*=12-17 per group). (A) Novel object recognition testing revealed there was an absence of statistically significant differences between wild-type (black circles), *Cps1*^N674I/N674I^ (blue squares) and *Cps1*^0/N674I^ (red triangles) mice. (B) In open field testing, measurement of entry to the center was reduced with increasing loss of Cps1. (C) Quantitative measurement of distance traveled was similarly reduced with increasing *Cps1* loss. Together, B and C suggest an anxiety-like behavior. (D-F) Light/dark transition testing further suggested anxiety-like behavior: number of entries to the lighted area (D), total amount of time spent in the light side (E) and average speed in the light (F) was reduced for the compound heterozygote. ns, not significant; *P<0.05, **P<0.01, ***P<0.001, ****P<0.0001 (A-F, one-way ANOVA with Dunnett's T3 multiple comparison test).

Novel object recognition was performed to assess recognition memory (Fig. 6A). Mice possess an innate predisposition for novelty (Antunes and Biala, 2012), having a propensity to explore a new object being quantified using the discrimination index. Both hypomorphic models on a 20% protein chow demonstrated similar discrimination indices to WT mice. These data indicate that recognition learning and memory in both *Cps1*^N674I/N674I^ and *Cps1*^0/N674I^ mice are without deficit.

Open field testing (Fig. 6B,C), a quantitative measurement of overall locomotor behavior, also measures mouse anxiety by assessing entry into the center and distance traveled during the first 5 min while the field is still a novel environment. Anxious animals tend to spend less time in the open area, have decreased exploratory behavior and prefer more time closer to the walls (Binder et al., 2004). WT mice averaged 23.8±7.1 center entries, *Cps1*^N674I/N674I^ averaged 18.5±7.7 center entries (*P*=0.103) and *Cps1*^0/N674^ averaged 15.2±9.0 center entries (*P*=0.021). Distance traveled in 5 min had a similar graded response: WT traveled 18.15±3.66 m, *Cps1*^N674I/N674I^ mice traveled 16.37±5.56 m (*P*=0.493) and *Cps1*^0/N674^ mice traveled 11.85±4.47 m (*P*=0.002).

Light/dark transition testing (Fig. 6D-F) is based on the innate aversion of mice to brightly lit areas and is conducted to observe whether an anxiety phenotype exists. Regarding entries into the brightly lit area (Fig. 6D), WT mice had 12.4±5.3 entries, *Cps1*^N674I/N674I^ had 10.4±4.0 entries (*P*=0.429) and *Cps1*^0/N674I^ had 5.8±3.0 entries (*P*=0.0009). Total time in the brightly lit area (Fig. 6E) was also reduced in a Cps1-graded manner: WT, 89.4±33.4 s; *Cps1*^N674I/N674I^, 84.1±28.7 s (*P*=0.862); and *Cps1*^0/N674^, 29.6±24.5 s (*P*<0.0001). Similarly, the average speed in the light was measured (Fig. 6F): WT had an average of 0.0395±0.008 m/s, whereas *Cps1*^N674I/N674I^ had an average of 0.044±0.006 m/s (*P*=0.182), and *Cps1*^0/N674^ had a significantly reduced average of 0.0225±0.014 m/s (*P*=0.003). Together, these data, along with the results from the open field assessment, suggest an anxiety-like phenotype in the *Cps1*^0/N674^ mice.

Results from additional behavioral testing when mice were receiving high-protein chow (novel object recognition, open field and light/dark transition testing) were not substantially different from the findings from mice with standard chow (Fig. S1). However, elevated plus maze demonstrated statistical trends, with reduced entries in (*P*=0.0965) and time in (*P*=0.1096) the open arms for *Cps1*^0/N674I^ mice compared to WT controls, consistent with an anxiety phenotype.

**Fig. 7.**
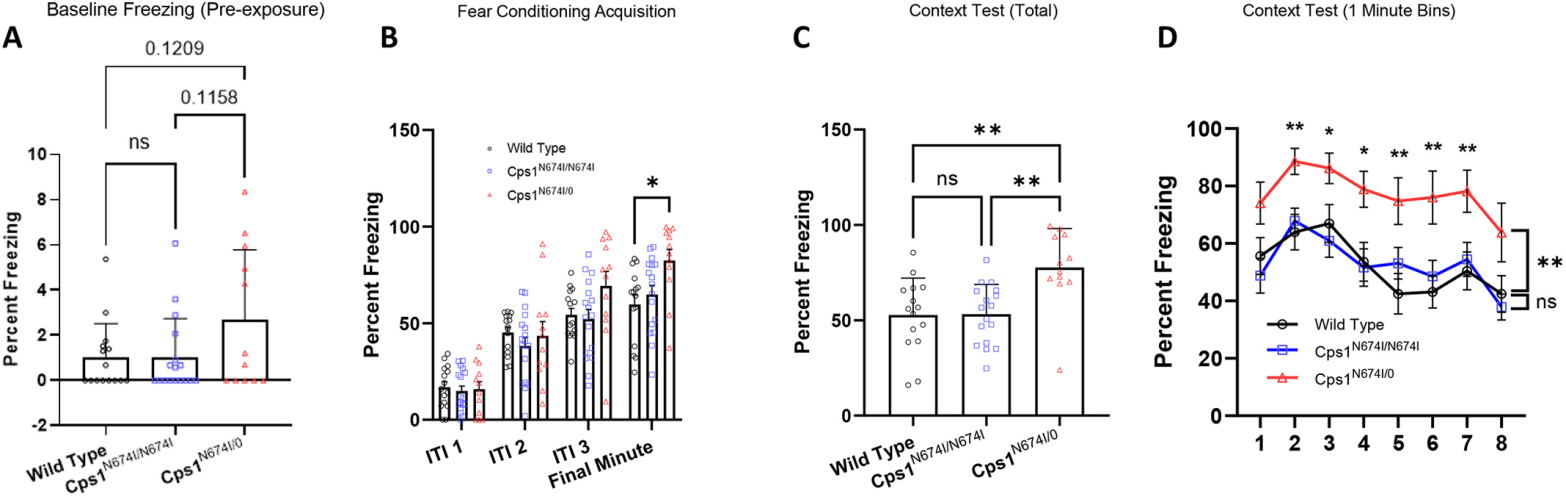
**Conditioned fear testing of hypomorphic compound heterozygote receiving high-protein chow further confirms an anxiety-like phenotype.** Behavioral phenotype testing was performed on adult mice of each genotype (*n*=12-17 per group). In fear conditioning studies of contextual memory in which sexes were combined for analysis, there was evidence of increased freezing in the compound heterozygote. (A) At baseline, freezing was increased in Cps1 compound heterozygote mice. (B) During acquisition, there were no differences during the acquisition (i.e. shock) intertrial intervals (ITIs), demonstrating that mice were freezing following shocks. The only difference was in the final minute of the third ITI as the compound heterozygote demonstrated increased freezing. (C,D) However, upon re-exposure to context 24 h later, total freezing (C) and freezing across the whole session (D) were increased in the compound heterozygote compared to homozygous mutant and wild-type controls, demonstrating that the compound heterozygous mice had stronger contextual fear conditioning memory, consistent with an anxiety-like phenotype. ns, not significant; *P<0.05, **P<0.01 (A,C, ordinary one-way ANOVA with Tukey's multiple comparison test; B,D, two-way ANOVA with mixed effects analysis).

Conditioned fear testing was performed to study associative learning between a foot shock and the environmental context, relative to fear and anxiety (Fig. 7). The dependent measure is a freezing response and was assessed pre-exposure to assess this activity at baseline (Fig. 7A). WT and *Cps1*^N674I/N674I^ mice showed similar levels of freezing. However, *Cps1*^0/N674^ mice demonstrated a trend towards increased freezing compared to WT (*P*=0.1209) and *Cps1*^N674I/N674I^ (*P*=0.1158). For acquisition, mice are placed in a chamber and shocked three times. Percentage freezing was then assessed during the shock intertrial intervals (ITIs). No statistically significant differences were found in baseline freezing or acquisition, except in the last minute of intertrial 3; here, *Cps1*^0/N674^ demonstrated statistically significantly (*P*=0.0151) increased freezing compared to WT (Fig. 7B). The subsequent day, context testing was performed (Fig. 7C), and although there was no statistically significant difference between WT and *Cps1*^N674I/N674I^ in total freezing, percentage total freezing was increased in *Cps1*^0/N674^ compared to WT (*P*=0.0029) and *Cps1*^N674I/N674I^ (*P*=0.0027). In addition, there was statistically significantly increased freezing across the whole session in the *Cps1*^0/N674^ mice. Together, these data suggest the Cps1 compound heterozygote mice have a heightened general anxiety state, rather than specific fear memory.

## DISCUSSION

Existing interventions poorly mitigate the effects of CPS1 deficiency, particularly in those completely lacking enzyme activity, and the outcomes are poor. As in humans, the prognosis for mice is also poor (Schofield et al., 1999; Khoja et al., 2019) as there are limited, if any, interventions that can be performed once suckling, milk is consumed and hyperammonemia begins. The severity of the disorder, the inability to develop and test therapies in a representative model not requiring postnatal somatic gene modification and the necessary 3- to 4-week gene ramp down before Cps1 protein depletion reaches critical levels (Khoja et al., 2018) led us to develop a new mouse model with longevity and mild to moderate hyperammonemia.

Examining the literature on known human mutations, specific details (e.g. CPS1 activity) are often lacking. Nonetheless, by examining compound heterozygous mutations in which one allele contains a nonsense mutation, we identified reasonable mutation candidates to consider. Still, not knowing how a mutation in a human would translate into a mouse model remained an open question. Of the cases in which such data were available, one stood out as potentially being a good candidate: a late-onset compound heterozygote (Kurokawa et al., 2007), where a 13-year-old girl presented with ammonia of 938 µg/dl (i.e. 550 µmol/l), low plasma citrulline and no intellectual disability. She possessed CPS1 variant c.2021A>T, p.Asn674I found with the nonsense variant c.1528delG, p.fs514*, having 17% hepatic CPS1 enzymatic activity.

The CPS1 Asn674Ile variant was predicted to be deleterious by four variant effect predictors. Isoleucine was also absent at position 674 in a multiple sequence alignment of over 50,000 CPS1 orthologs. In addition, amino acids with hydrophobic side chains (i.e. isoleucine, a nonpolar side chain, more likely to be found in the interior of proteins) are typically absent or under-represented at that residue in the bicarbonate phosphorylation domain of CPS1, albeit outside of the active center. Protein structure and folding were predicted to have a minimal difference from the native protein. Therefore, based on modeling and the existing human findings, we reasoned that Asn674Ile would be a mild pathogenic variant in mouse, resulting in phenotype consistent with the clinical phenotype of the late-onset compound heterozygote female, e.g. survival, elevation in ammonia and consequent mild amino acid derangements. This proved to be true.

However, there is a lack of clarity in exactly the dysfunction that Asn674Ile is causing with Cps1 expression and function. Based on structure–function domains of the protein, this mutation occurs in the bicarbonate phosphorylation region. In this three-step reaction in the C-terminal moiety, it is not clear whether the mutation is causing dysfunction at step 2 in the production of carbamate or there is an issue with carboxyphosphate production in step 1. Spatially, the N674 residue is located some distance away from the loops that bind to ATP and stabilize the formation of carbamate. The N674 residue is closer to the glutaminase-like domain of CPS1, whereas other loops are located closer to the underside of the domain. As it is not near the ends of the exon, and SpliceAI does not predict this variant to be a splice variant, we do not believe that this is leading to a truncated protein. Instead, the protein in both the homozygous mutant and the compound heterozygote is reduced in abundance, resulting in altered overall activity under both unstrained living conditions and with alterations in protein intake (i.e. increased protein in diet) or ammonium loading. Gene variants, in general, can interfere with cellular steady-state protein abundance by multiple different mechanisms: diminished thermodynamic stability, altered post-transcriptional regulation and interrupted trafficking, to name a few (Matreyek et al., 2018; Yue et al., 2005). Indeed, it has been reported that up to 75% of pathogenic variations that cause monogenic disease are believed to disturb thermodynamic stability and subsequently alter abundance; missense mutations, in particular, are more likely to occur at genomic positions that alter the native protein's structural stability (Redler et al., 2016). In aggregate, our findings and the previous studies referenced, while speculative, suggest that the mutated full-length Cps1 protein produced is functional, but that this missense variant, and the resulting amino acid substitution, disturbs the native stability of the protein, and, consequently, this destabilized protein is reduced in abundance.

A pattern of plasma metabolic amino acid derangement occurs in CPS1 deficiency. Ammonia accumulates, with shunting to alternative metabolic pathways subsequently occurring. Glutamate synthesis from ammonia and alpha-ketoglutarate typically results in increased glutamate levels. In addition, excess ammonia may drive the conversion of glutamate to glutamine, resulting in plasma elevation of both glutamate and glutamine. Elevated levels of ammonia also affect alanine, a key amino acid involved in the transfer of nitrogen from peripheral tissues to the liver by conversion from pyruvate. Glycine production may increase as it can serve as a carrier of nitrogen, as may asparagine, being synthesized from aspartate and glutamine. As CPS1 is responsible for production of carbamoyl phosphate, there is a decrease in such levels, resulting in a decline in citrulline synthesis. Catabolism of other amino acids, particularly ones related to the urea cycle, may occur as excess nitrogen is managed. As part of this, lysine and the aromatic amino acids can also be deranged.

Both hypomorphic models demonstrate elevated ammonia, have altered responses to ammonium, and possess reduced Cps1 protein and enzyme activity at ∼20% of normal (remarkably similar to the mutation's effect in the described human patient); reduced citrulline and arginine with elevated aspartate and glutamate also occur in the compound heterozygote, further mirroring human CPS1 deficiency. Furthermore, both hypomorphic models have a decreased flux through the urea cycle when compared to WT, as shown by reduced enrichment of labeled urea and citrulline. Both showed non-statistical responses to the administration of carglumic acid, likely owing to endogenous NAGS already being upregulated, resulting in endogenous N-acetylglutamate enhancing feedback by activating the biosynthesis of carbamoyl phosphate by Cps1. Cps1 expression, elevated in the homozygous mutant but not in the compound heterozygote, is likely due to there being only one allele to express Cps1, the other being a null mutation. Although the models do differ from neonatal deficiency patients who exhibit severe CPS1 deficiency at birth, they capture important aspects of the disorder, particularly for the later-onset patient with mild to moderate enzymopathy. Importantly, these new Cps1 mouse models, noting particularly the compound heterozygote, represent usable systems to study therapies for CPS1 deficiency, as the mice phenocopy many of the biochemical aspects of the disorder, including elevated ammonia and the typical amino acid derangements, albeit mild in magnitude. Importantly, these mice are of normal size, have no underlying anatomic liver abnormality, have normal transaminases and are fertile. Moreover, the phenotype is highly reproducible and fully penetrant. Most of all, the residual Cps1 enzyme activity, which is functional, is comparable to that of patients with later-onset CPS1 deficiency. Together, these models are an advance over the neonatal-lethal constitutive Cps1 mouse (Khoja et al., 2019) and the conditional Cps1 knockout (Khoja et al., 2018) previously developed.

The Asn674Ile homozygote and the compound heterozygote have chronic mild hyperammonemia (that increases with high-protein chow) and demonstrate findings of anxiety on multiple behavioral tests. This is the not the first time a relationship between chronic hyperammonemia and anxiety-like behavioral in rodents has been detected. We had previously found persistently elevated ammonia in mice when developing a gene therapy approach for CPS1 deficiency (Diep et al., 2024); those studies also demonstrated the presence of anxiety-like behavior. In other studies, elevated ammonia was found to cause anxiety-like behavior in fish (Zhang et al., 2023), and open field exploratory behavioral testing of bile duct-ligated rats with chronic hyperammonemia revealed activity with less time in the center and shorter travel distances consistent with anxiety-like behavior (Leke et al., 2012). This latter model, however, was induced with biliary cirrhosis in mature rodents, and the *CPS1* gene therapy studies were conducted in adult mice, both with already developed nervous systems. These new models allow for probing of aspects of nervous system development in the face of chronically elevated ammonia since birth.

In conclusion, two new murine models of CPS1 deficiency have been developed that demonstrate biochemical and enzymatic evidence of mild to moderate Cps1 deficiency. Both models are long-lived, and demonstrate at least mildly elevated ammonia, impaired flux through the urea cycle, altered metabolic response to ammonium loading and increases in protein intake. Such models could assist in the development of new therapies for CPS1 deficiency and be used in probing the effects of chronic mild hyperammonemia on brain development.

## MATERIALS AND METHODS

### CPS1 variant analysis

Pre-calculated SIFT, PolyPhen2, AlphaMissense and EVE variant effect predictions for human CPS1 Asn674Ile were accessed from Ensembl Variant Effect Predictor tool (McLaren et al., 2016). The multiple sequence alignment of 50,951 CPS1 orthologs was accessed from EVE on 25 April 2024 (Frazer et al., 2021) to calculate the number of each variant at residue 674.

The structures of human and murine WT CPS1 and Asn674Ile variants were predicted using AlphaFold3 with auto seed via the AlphaFold server (Abramson et al., 2024). Input protein sequences are based on NP_001866.2 and NP_001074278.1 and exclude the mitochondrial transit peptide (amino acids 1-38 for human and mouse CPS1). AlphaFold3 jobs include two entities of ADP. The top-ranked predictions were further visualized and analyzed in PyMOL 1.8.2 (The PyMOL Molecular Graphics System, Version 1.2r3pre, Schrödinger, LLC). Protein structure alignments was performed using align function in PyMOL and TM-align server (Zhang and Skolnick, 2005). The distance between the centroids of Asn674 and ADP in the bicarbonate phosphorylation domain was measured in PyMOL using the distance function.

*In silico* protein analysis to assess for probability of the Ans674Ile undergoing protein truncation was performed for NM_001875.5(CPS1):c.2021A>T (p.Asn674Ile) for hg38 at VCV002577361.1 (ClinVar, NCBI). The variant was entered into SpliceAI to obtain the probability that the variation results in acceptor gain/loss and donor gain/loss scores.

### KI mouse development

KI mouse development occurred at the Mouse Development Program at the University of California, Davis (UC Davis). All animal work was done under protocols approved by the UC Davis International Animal Care and Use Committee, and animals were housed under veterinary oversite, ensuring compliance with federal and state regulations, inspecting animal facilities and laboratories, and overseeing training and educational programs. UC Davis is Association for Assessment and Accreditation of Laboratory Animal Care accredited (#000029) and maintains Public Health Service assurance [D16-00272 (#A3433-01) and US Department of Agriculture Registration (#93-R-0433)].

#### CRISPR-based modification design

**Fig. 8.**
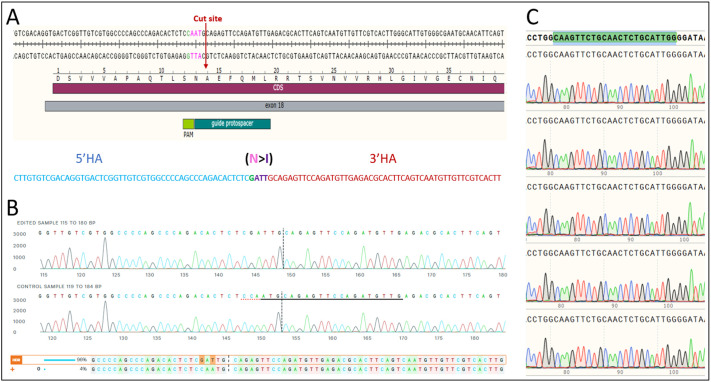
**CRISPR design showing the endogenous exon 18 of *Cps1* and genetic development of Asn674Ile Cps1 hypomorphic mouse model.** (A) Pink AAT is the N codon, green C is the silent mutation target to ablate the protospacer adjacent motif (PAM), red cut site shows the cleavage point. The dark green is the gRNA protospacer, light green is the PAM. The sequence of the single-stranded oligodeoxynucleotide repair template shows the 5′ homology arm (blue), 3′ homology arm (red), the introduction of ATT (N>I) and the silent mutation (green). (B) Chromatogram alignment between the identified founder (top) and the wild-type control (bottom). Bottom sequence report shows a 96% contribution of homology-directed repair allele (Ans674Ile) per inference of CRISPR edits (ICE) analysis output. (C) Chromatogram alignment from N1 animals of the potential off-target from chr1:155673955-155673977, showing the region as intact and without off-target indels.

The *Cps1* mouse ortholog from mm39 was compared to human *CPS1*, which was found to be 95% identical and specifically with the asparagine (Asn or N) both located at residue position 675 between species. A single-guide RNA (gRNA), CAACATCTGGAACTCTGCAT(TGG), was identified using the online tool CRISPOR and assessed for predicted targeting efficiency as well as potential off-targets in the mouse genome (Concordet and Haeussler, 2018). Predicted cleavage site for this gRNA is one nucleotide downstream of the desired Ans674Ile modification, making it a prime candidate gRNA for homology-directed repair (HDR) using a single-stranded oligodeoxynucleotide (ssODN) as a repair template. The ssODN, CTTGTGTCGACAGGTGACTCGGTTGTCGTGGCCCCAGCCCAGACACTCTCGATTGCAGAGTTCCAGATGTTGAGACGCACTTCAGTCAATGTTGTTCGTCACTT, contained 50 nucleotide homology arms, the AAT to ATT (Asn to Ile) codon modification, as well as a silent mutation in the upstream serine (TCC to TCG) to ablate the protospacer adjacent motif site to prevent further cleavage of the HDR allele (Fig. 8A). A potential off-target site was identified to have an MIT off-target score greater than 1.5 (2.34257) showing a three-mismatch protospacer hit at chr1:155673955-155673977 (mm39), for which we designed PCR/sequencing primers for screening of animals in addition to on-target assays (see doi:10.6084/m9.figshare.29260724 titled offtargets_mm39-chr1-67211850-67211873-1.xls).

#### Gene targeting

Upon receipt, the CRISPR RNA (crRNA) was tested and confirmed *in vitro* by ribonucleic protein (RNP) cleavage analysis against a purified PCR product containing the target sequence. C57BL/6J zygotes were harvested and electroporated as described previously (Chen et al., 2016). Briefly, CRISPR Cas9 prep included Alt-R *S.p.* Cas9 Nuclease V3 complexed with a two-part gRNA (tracr:crRNA) in a 1:1.25 molar ratio, resulting in a 16 μM RNP. Addition of ssODN repair template with 20 μM concentration was included in the final electroporation prep [Integrated DNA Technologies (IDT), Coralville, IA, USA]. Approximately 80 zygotes underwent electroporation and were transferred to oviducts of CD-1(ICR) pseudopregnant females. Subsequent P0 pups were tail clipped at weaning, and DNA was extracted using DNAdvance magnetic beads (Beckman Coulter, Brea, CA, USA) on a LIMS integrated Hamilton STAR liquid handler (Hamilton Robotics, Reno, NV, USA). DNA were PCR amplified with oligonucleotides (fwd, 5′-TTAATCCTTTCCTCGTGTCTTCG-3′; rev, 5′-GCAATCATTCTGATCTTACCCAGT-3′), resulting in 347 bp WT/KI band as well as other indel-induced alternate band sizes. Amplicons were PCR purified using a Monarch PCR & DNA Cleanup Kit (New England Biolabs, Ipswich, MA, USA; T1030S) and submitted to Sanger sequencing (Azenta Life Sciences, Burlington, MA, USA). Subsequent sequence files were analyzed using the Synthego ICE analysis online tool (Conant et al., 2022). From 16 pups born, we identified a nearly homozygous HDR male founder that was set to backcross with stock C57BL/6J mice to transmit the Cps1_Asn674Ile allele in the germline (Fig. 8B). Subsequent N1 generation animals underwent PCR and were sequenced in the same manner as the P0 founder animals, with 80% (*n*=15) of the N1 animals sequencing as heterozygous. The potential off-target locus identified during CRISPR-based KI design was PCR amplified with primers (fwd, 5′-CTTCAACCTCTGCCTGCAC-3′; rev, 5′-AAATTCGGGCTTTCACATTG-3′) and submitted to Sanger sequencing (Azenta Life Sciences) for all N1 heterozygous animals. The potential off-target locus was the only off-target of concern based on the MIT score and harboring only three mismatches within the protospacer, in accordance with recently published CRISPR off-target assessment (Peterson et al., 2023). Chromatograms were aligned and confirmed to not contain unintentional indels at chr1:155673955-155673977 (Fig. 8C). N1 mice were further mated to C57BL/6J breeders and derived N2 heterozygous mice.

### Animal care and mouse procedures

Experimental procedures were approved by and conducted according to the guidelines of the University of California, Los Angeles (UCLA) Chancellor's Animal Care and Use Committee. Mice were housed in conventional cages with vented lids in a vivarium at UCLA and kept according to the National Institutes of Health guidelines with temperature and humidity control. Water and standard mouse chow with 20% protein (PicoLab Rodent Diet 20, 5053) were consumed *ad libitum*, and mice were maintained on a 12-h light–dark cycle. Higher-protein diet (52% protein) was custom prepared by TestDiet (Newco Distributors, Rancho Cucamonga, CA, USA; 5TVW). To generate homozygous Asn674Ile mice, random brother/sister inbreeding was performed. To generate compound heterozygotes *Cps1*^N674I/0^, homozygous *Cps1* Asn674Ile mice were mated with heterozygote exon 3-4 *Cps1* mice (Khoja et al., 2019). Drinking water, where indicated, was prepared with 0.1% carglumic acid (Sigma-Aldrich, St Louis, MO, USA; 1188-38-1) and 5% dextrose (Sigma-Aldrich, G8270-10KG). Characterization studies were performed on mice 12-16 weeks of age.

### Genotyping

#### Exon 3-4 null PCR genotyping

Exon 3-4 genotyping was determined by traditional PCR with amplicons run on a 2% agarose gel, with genomic DNA prepared from ear clips by standard methods. This involved 35 cycles of PCR (initiation/melting, 98°C×3 min; one cycle); denaturation (35 cycles; step 1, 98°C×15 s; step 2, 60°C×30 s; step 3, 72°C×40 s); then 72°C×5 min. Mutant band was visualized at 600 bp and the WT band at 400 bp.

Primers were as follows: *Csd1* F, 5′-AGGGTTGTATGCTTCATCTTGTATGC-3′; *Csd1* ttr, 5′-AGAAAAGCTGGTGCTCATACAAAGG-3′; *Csd1* R, 5′-CCACTAATGGATGGAATGACTATGTGC-3′; *Csd1* Lac F, 5′-GCTACCATTACCAGTTGGTCTGGTGTC-3′.

#### Asn674Ile PCR genotyping

Asn674Ile genotyping was determined by real-time quantitative PCR. Genomic DNA was prepared from ear clips by standard methods. This involved 40 cycles of PCR (initiation/melting, 95°C×15 min; one cycle); denaturation (40 cycles; step 1, 95°C×30 s; step 2, 60°C×60 s).

Primers were as follows: *Tcrd* F, 5′-CAGACTGGTTATCTGCAAAGCAA-3′; *Tcrd* R, 5′-TCTATGCCAGTTCCAAAAAACATC; *Tcrd* MGB VIC Probe, 5′-VIC-ATTATAACGTGCTCCTGG-MGB-3′; TM_Cps1-F, 5′-GACAGGTGACTCGGTTGTC-3′; TM_Cps1-R, 5′-CCAAGTGACGAACAACATTGAC-3′; *Cps1* MGB FAM Probe, 5′-Fam-AACTCTGCAATCGAG-MGB-3′.

### Measurement of whole-blood ammonia

Whole-blood ammonia (from retroorbital collection) was determined (with dilutions as needed) using a PocketChem BA meter (Arkray, Kyoto, Japan; PA-4140).

### Ammonia challenge

Non-fasted mice underwent ammonia challenging generally between 09:00 and 11:00, being injected by intraperitoneal method with a 5 mmol/kg solution of NH_4_Cl (Cambridge Isotope Laboratories, Andover, MA, USA; NLM-467-1) as previously described (Diep et al., 2024; Ye et al., 1997). Behavioral scores were based on the following: ataxia (0-2), seizure activity (0-2) and response to sound (0-3) (Ye et al., 1997).

### Quantitative reverse transcription PCR for RNA expression

Tissue samples were collected from mice, and total RNA was extracted from livers using an RNeasy Fibrous Tissue Mini Kit (Qiagen, Hilden, Germany; 74704) according to the manufacturer's instructions. Then, 1 μg RNA was reverse transcribed using a High Capacity cDNA Reverse Transcription Kit (Applied Biosystems, Waltham, MA, USA; 4368814).

Following cDNA generation, real-time PCR (qPCR) was performed using SsoAdvanced Universal SYBR Green Supermix (Bio-Rad, Hercules, CA, USA; 1725274) to quantify the expression of the *Cps1*, *Nags* and β-actin genes using three sets of primer pairs. Melting temperatures for the primer sets were 56°C, 58°C and 56°C, respectively. Quantification was determined via threshold cycle (C_T_) values using a MyiQ2 Two Color Real-Time PCR Detection System (Bio-Rad, 170-9790). The qPCR protocol using the *Cps1* primers was performed as follows: DNA denaturation for 3 min at 95°C, primer annealing for 10 s at 95°C, then primer extension for 25 s at 56°C. The qPCR protocol using the *Nags* primers was performed as follows: activation at for 2 min at 50°C, DNA denaturation for 10 min at 95°C, primer annealing for 15 s at 95°C, then primer extension at 60°C for 60 s. The primer annealing and primer extension steps for all primer sets were repeated for up to 40 cycles. *Cps1* and *Nags* expression were normalized to endogenous β-actin and fold enrichment of the *Cps1*^N674I/N674I^ and *Cps1*^0/N674I^ groups was calculated using the 2^−ΔΔCt^ in comparison to the WT group.

Primers used were as follows: *Cps1* F, GCTCTATCGACCTTGTTATCA; *Cps1* R, AGTGAAACAGTGACTTGCTA; *Nags* F, GCCTGCGGAATAACAGTCAGAAG; *Nags* R, TCCACGATGAGCCGAATCTGCT; β-actin F, CTAAGGCCAACCGTGAAAAG; β-actin R, ACCAGAGGCATACAGGGACA.

### Western blot analysis of Cps1 protein

Frozen liver (stored at −80°C until use, unfixed) was sonicated with soluble protein isolated in RIPA buffer (Thermo Fisher Scientific, Waltham, MA, USA; 89900) with HALT protease inhibitor (Thermo Fisher Scientific, 78430). Protein quantification was performed using protein assay dye (Bio-Rad, 5000006) and loaded into a 12% gel (Bio-Rad, 4561045). This was followed by transfer to PVDF membranes using a TransBlot Turbo system according to the manufacturer's instructions (Bio-Rad, 1704156). Blocking in 5% milk in PBST for 1 h at room temperature (RT) was performed, followed by incubation with primary antibodies overnight at 4°C with gentle agitation. Secondary antibodies were incubated for 1 h at RT; detection was then performed with SuperSignal West Pico PLUS Chemiluminescent Substrate (Thermo Fisher Scientific, 34577). Primary antibodies were as follows: anti-CPS1 (Bio-Rad, RM395, used at 1:1000); anti-β-actin (Santa Cruz Biotechnology, Santa Cruz, CA, USA; sc-47778, used at 1:1000). Secondary antibodies were as follows: goat anti-rabbit IgG H&L (HRP) (against CPS1 primary; Bio-Rad, 1706515, used at 1:5000); goat anti-mouse IgG H&L (HRP) (against β-actin primary; Bio-Rad, 1706516, used at 1:5000). Blots were then imaged using an iBright FL1500 imaging system (Invitrogen, Waltham, MA, USA; A44241) and quantified with their proprietary software. iBright Analysis Software was used to acquire densitometry data by subtracting the background intensity from the volume for each band. The rolling background correction volume was used for the final densitometry measurements. The rolling background correction volume of each Cps1 band was taken as a percentage of its respective internal positive β-actin band. The average percentage volume for the WT samples in the blot was then calculated, and each sample was standardized to this average. These relative percentage volume values were then plotted and analyzed for significance.

### Immunohistochemistry, microscopy image acquisition, intensity comparison

Mice were perfused transcardially with ice-cold 1× PBS and 4% PFA, fixed and paraffin embedded by standard techniques. Wet heat-induced epitope retrieval was carried out as previously described (Krenacs et al., 2010). Sections were permeabilized with 0.3% Triton X-100 (MilliPore Sigma, Saint Louis, MO, USA; T9284), and nonspecific binding was blocked by 10% NGS (Thermo Fisher Scientific, 10000C) with 0.3% Triton X-100 and incubated overnight at 4°C with primary antibodies: anti-CPS1 (Bio-Rad, RM395, used at 1:250), anti-glutamine synthetase (Thermo Fisher Scientific, MA5-27749, used at 1:250). Following PBS washes, host-conjugated secondary antibodies were incubated at RT for 2 h [goat anti-rabbit Alexa Fluor 594 against CPS1 antibody (Thermo Fisher Scientific, A-11012, used at 1:500), goat anti-mouse Alexa Fluor 488 against glutamine synthetase antibody (Thermo Fisher Scientific, A-11001, used at 1:500) and PureBlu DAPI (Bio-Rad, 1351303, used at 1:500)]. After washes, sections were mounted with ProLong Gold Antifade Mountant (Thermo Fisher Scientific, P36934) and coverslipped.

16-bit images were acquired on an inverted Leica DMi8 fluorescence microscope (DAPI, GFP, Cy3 and Cy5 filters; Leica Microsystems, Wetzlar, Germany). Tiled liver images (20×/0.8 NA HC Plan-Apochromat DRY objective; Zeiss, 115006530) were mosaic merged (7% overlap) and processed on Leica LAS X microscopy software (version 3.7.6.35997) using DAPI nuclei stain as the reference focus to standardize depth of field. Additionally, exposure times and intensity range (minimum as 0, maximum as 65,536 gray) for each fluorescence channel was kept consistent between images.

CPS1 mean intensity for each of the 16-bit images, based on gray-scale values, was calculated by averaging five random regions of interest (ROIs) of the same size and relative location within the intensity range chosen for CPS1 (red, 594 nm) (see Fig. S2). Absolute values were also converted to percentages (%). Microscopy images are presented with an over-/under-exposure filter displaying the five ROIs and as merged fluorescent images (DAPI, blue; CPS1, red; glutamine synthetase, green).

### Plasma and tissue amino acid measurement

Liver tissue (200 mg) was homogenized in 1 cm^3^ Seraprep (Pickering Laboratories, Mountain View, CA, USA; SP100) and centrifuged at 20,000 ***g*** at 4°C for 10 min. The supernatant was removed and treated the same as plasma for amino acid analysis. Approximately 30-100 µl sample was mixed with sample dilution buffer Seraprep and internal standard (S-2-aminoethyl-1-cysteine). Next, the mixture was centrifuged for deproteinization. Filtered supernatant was injected to a L-8900 Amino Acid Analyzer (Hitachi, Tokyo, Japan). In the amino acid analyzer, individual amino acids were separated by ion-exchange liquid chromatography. Amino acid effluents reacted with ninhydrin, resulting in color products, which were measured with visible spectrophotometry at wavelengths of 440 and 570 nm. The chromatogram was integrated and quantitated with EZchrome software (Agilent Technologies, Santa Clara, CA, USA) by comparing sample signal intensities with standard amino acids of known concentrations.

### Ureagenesis

After collection of a baseline blood sample obtained retro-orbitally under anesthesia, ^15^NH_4_Cl (≥99% isotope enrichment, from Cambridge Isotope Laboratories, Tewksbury, MA, USA) was injected intraperitoneally at a dose of 4 mmol/kg. After 30 and 60 min, another blood sample was collected prior to ureagenesis analysis using an assay modified from a published method (Allegri et al., 2017, 2019). Collected samples were stored at −20°C until analysis. Isotopic ratios were measured as the fraction of urea or amino acids in the sample that became isotopically labelled ([^15^N]urea, [^15^N]citrulline, [^15^N]glutamine) normalized to the respective metabolite pool size, and the area under the curve for each metabolite from 0 to 60 min was calculated.

### ALT and AST determination

ALT and AST were determined in plasma from venous blood collected by capillary tubes with sodium heparin (Globe Scientific, Mahwah, NJ, USA; 56186) and processed at IDEXX Veterinary Services reference laboratory (Westbrook, ME, USA).

### Hepatic CPS1 enzyme activity assay

The determination of hepatic CPS1 enzymatic activity was performed similarly to as previously described (Makris et al., 2023).

### Behavioral testing

Behavioral tests were performed during the light cycle (06:00-18:00) between the hours of 12:00 and 17:00. To reduce handling stress during testing, all study mice were handled by the experimenters for 5 days prior to any behavioral testing. To acclimate mice to the environment, all mouse home cages were placed in the behavior room for at least 1 h prior to testing. Experimenters were unaware of the treatment conditions.

SHIRPA, open field, novel object recognition and contextual fear conditioning were performed as previously described (Diep et al., 2024). Light/dark transition testing was performed as described previously (Kulesskaya and Voikar, 2014; Takao and Miyakawa, 2006).

### Statistical analysis

Collected data were analyzed with a statistical software package (GraphPad Prism 10). All results were expressed as mean±s.d. *P*-values were determined using one-way ANOVA with Dunnett's T3 multiple comparisons test, ordinary one-way ANOVA with Tukey's multiple comparison test, two-way ANOVA with mixed-effects analysis, or paired two-tailed *t*-test with Welch's correction, when applicable [ammonia response to carglumic acid, paired two-tailed *t*-test; behavioral testing, novel object recognition and open field test, one-way ANOVA; fear conditioning, two-way ANOVA; SHIRPA, one-way ANOVA]. For immunostaining, one-way ANOVA against the WT group was performed on absolute intensity values and percentages. Post hoc multiple comparisons were assessed using Dunnett’s T3 hypothesis testing. *P*<0.05 was considered significant.

## Supplementary Material

10.1242/dmm.052303_sup1 Supplementary information
