# Supplementary material for: A hypomorphic model of CPS1 deficiency for investigating the effects of hyperammonemia on the developing nervous system
Source: Dis Model Mech. 2025 Jun 20;18(7):dmm052303. doi: 10.1242/dmm.052303 (PMC12208401; doi:10.1242/dmm.052303)
Supplement: Supplementary information [file dmm-18-052303-s1.pdf]

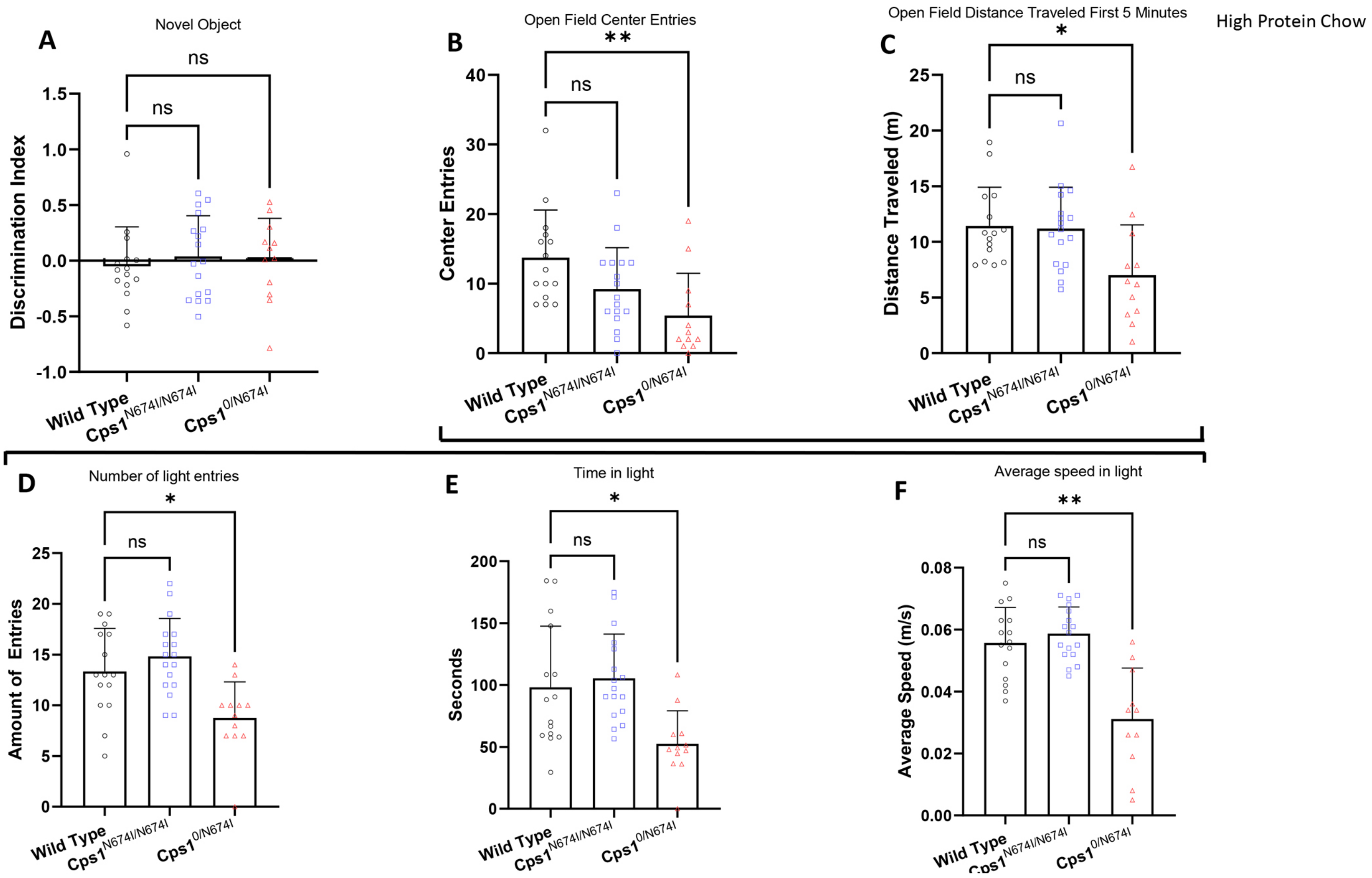

Fig. S1. Behavioral testing of hypomorphic compound heterozygote receiving high protein mouse chow further confirms an anxiety-like phenotype. Behavioral phenotype testing was performed in adult mice of each genotype (n = 12-17 per group). **A)** Novel object recognition testing revealed there was an absence of statistically significant differences between wild type (black circles), Cps1$^{N674I/N674I}$ (blue squares) and Cps1$^{0/N674I}$ (red triangles) mice. In open field testing, measurement of entry to the center (**B**) was reduced with increasing loss of Cps1. Quantitative measurement of distance traveled (**C**) was similarly reduced with increasing Cps1 loss. Together B and C suggest an anxiety-like behavior. Light dark transition testing (**D – F**) further suggested anxiety-like behavior: **D)** Number of entries to the lighted area, **E)** total amount of time spent in the light side, and **F)** average speed in the light was reduced for the compound heterozygote (* = $p < 0.05$, ** = $p < 0.01$, *** = $p < 0.001$, **** = $p < 0.0001$, ns = not significant.)

Disease Models & Mechanisms • Supplementary information

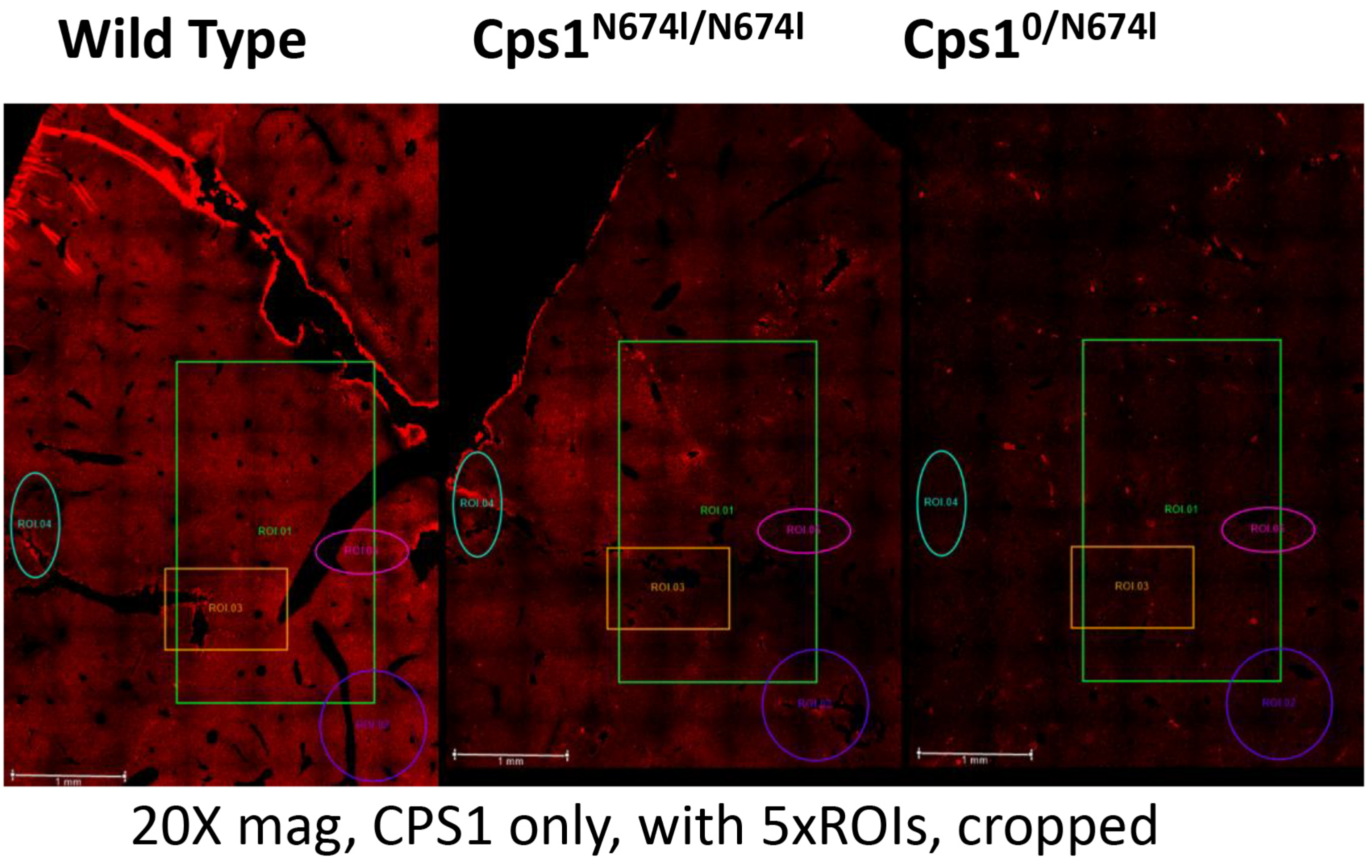

Fig. S2. CPS1 mean intensity for each of the 16-bit images, based on gray-scale values, was calculated by averaging five random regions of interest (ROIs) of the same size and relative location within the intensity range chosen for CPS1 (red, 594 nm) (see also Fig. 3E). Absolute values were also converted to percentages (%). One-way ANOVA against the WT group were performed on absolute intensity values and percentages. Post hoc multiple comparisons were assessed using Tukey's post hoc multiple comparison test.

Disease Models & Mechanisms • Supplementary information

Table S1. Ensembl variant effect and SpliceAI predictions of CPS1 Asn674Ile.

**Uploaded variant**	NC_000002.12:210606769:A:T
**Location**	2:210606770-210606770
**MANE Select Transcript**	NM_001875.5
**Ensembl Canonical Transcript**	ENST00000233072.10
**Exon**	18/38
**cDNA Position**	2156
**CDS Position**	2021
**SIFT (Score)**	Deleterious (0)
**PolyPhen (Score)**	Probably damaging (0.998)
**EVE Class (Score)**	Pathogenic (0.9047140320879404)
**AlphaMissense Classification (Pathogenicity Score)**	Likely pathogenic (0.7434)
**SpliceAI**<br>**Predicted Acceptor Gain Position (Delta Score)**<br>**Predicted Acceptor Loss Position (Delta Score)**<br>**Predicted Donor Gain Position (Delta Score)**<br>**Predicted Donor Loss Position (Delta Score)**	 14 (0.01)<br>-10 (0.00)<br>14 (0.00)<br>23 (0.00)

Disease Models & Mechanisms • Supplementary information

Table S2. Variant count in EVE multiple sequence alignment of CPS1 orthologs for residue 674.

Variant	Count
-	190
A	92
C	0
D	44918
E	560
F	0
G	21
H	15
I	0
K	193
L	4
M	0
N	4479
P	194
Q	189
R	16
S	38
T	22
V	4
W	0
X	11
Y	5

Disease Models & Mechanisms • Supplementary information
